# Mathematical analysis of fractional-order Caputo’s derivative of coronavirus disease model via Laplace Adomian decomposition method

**DOI:** 10.1186/s43088-022-00326-9

**Published:** 2022-12-06

**Authors:** Akeem O. Yunus, Morufu O. Olayiwola, Kamilu A. Adedokun, Joseph. A. Adedeji, Ismaila A. Alaje

**Affiliations:** 1 Department of Mathematic and Statistics, Osun State College of Technology, Esa-Oke, Osun State Nigeria; 2 Department of Mathematical Sciences, Faculty of Basic and Applied Sciences, Osun State University, Osogbo, 210001 Osun State Nigeria

## Abstract

**Background:**

The world's survival ability has been threatened by the COVID-19 outbreak. The possibility of the virus reemerging in the future should not be disregarded, even if it has been confined to certain areas of the world after wreaking such havoc. This is because it is impossible to prove that the virus has been totally eliminated. This research attempts to investigate the spread and control of the COVID-19 virus in Nigeria using the Caputo fractional order derivative in a proposed model.

**Results:**

We proposed a competent nine-compartment model of Corona virus infection. It starts by demonstrating that the model is epidemiologically sound in terms of solution existence and uniqueness. The basic reproduction threshold R_0_ was determined using the next-generation matrix technique. We applied the Laplace-Adomian decomposition method to the fractional-order Caputo's derivative model of the Corona virus disease to produce the approximate solution of the model analytically. The obtained results, in the form of an infinite series, were simulated using the MAPLE 18 package to investigate the effect of fractional order derivative on the dynamics of COVID-19 transmission in the model and shed light on methods of eradication. The graphical interpretations of the simulation process were shown and discussed accordingly.

**Conclusions:**

The study reveals the effect of the Caputo fractional order derivative in the transmission dynamics of the disease. Individual recovery was found to be greatest at an integer order, which represents the full implementation of other factors such as treatment, vaccination, and disease transmission reduction. Hence, we advised that researchers, government officials, and health care workers make use of the findings of this study to provide ways in which disease transmission will be reduced to a minimum to stop the prevalence of COVID-19 by applying the findings of this study.

## Background

The fatal Corona virus first emerged from Wuhan, China, before the end of 2019. Severe Acute Respiratory Syndrome Coronavirus 2 (SARS-CoV-2) was found to be the virus responsible for the illness, according to a medical agent’s investigation, and the World Health Organization named the virus-caused ailment Coronavirus Disease 2019 (COVID-19) (WHO, 2020b). As of July 2020, there had been more than 213 countries where this illness had spread, resulting in a total of 15,969,465 infections and 643,390 fatalities. The illness takes two to fourteen days to develop, and around 97.5 percent of affected people reportedly show symptoms within days [1–4]. As of June 4th, 2020, there were 11,516 confirmed cases in Nigeria, with 323 fatalities [5]. Numerous authors have anchored research relating to the transmission dynamics of COVID-19. To mention a few, research was presented in [6, 7], where the dynamical behaviors of nonlinear and linear COVID-19 transmission dynamics were studied. In [8], research was conducted on the construction of a mobile crowdsourced COVID-19 vulnerability map with geo-indistinguishability. They propose a novel approach to effectively construct a reliable community-level COVID-19 vulnerability map based on mobile crowdsourced COVID-19 self-reports without compromising participants’ location privacy, and the extensive simulations of their results based on real-world data demonstrate the proposed scheme's superiority over the peer designs in terms of estimation accuracy and reliability.

Mathematical modeling may be used to track the transmission of infectious illnesses. When followed, the use of mathematical principles and theory, as well as the work flow, strategy, and expected results, can end the course of illnesses. As a result, the biological sciences have become more reliant on mathematical modeling in recent years.

To analyze the progression of illnesses, a number of scholars have put forth various mathematical models. For instance, [9] explored a deterministic model of an infectious disease. The model was subjected to a numerical procedure based on the differential transform (DTM). The comparison between DTM and RK4 demonstrates how trustworthy DTM is for resolving epidemic models. A smoking model was proposed by the authors in [10]. Laplace Adomian Decomposition was used to examine the model's solution, which converges quickly to the specified precise solution. Numerical simulations of the model were also run to better understand how the parameters responded. The susceptible, infected, and recovered epidemic model was solved in a study reported in [11] using a powerful technique based on the generalized Taylor series, known as the residual power series approach. Convergence of the analytical approach for the SIR model solution is in the form of a power series. The graphical outcomes further illustrated the versatility of the residual power series strategy in solving a wide range of real-world issues involving differential equations of any order. A mathematical analysis of the rabies virus was done in [12]. The authors claimed that it is difficult to discover an exact solution for the model; therefore, they used the homotopy perturbation approach to achieve an approximation of the answer and compared their results to those of the Runge–Kutta fourth-order method.

Numerical modeling is crucial for forecasting the progression of illnesses. To simulate these proposed mathematical models, it is frequently necessary to obtain their solutions. Mathematicians frequently use numerical approximation techniques and transformation procedures, such as the Laplace Adomian decomposition method pioneered by [13], to achieve the analytical solution of complex mathematical models because it is challenging and occasionally impossible to do so.

For instance, researchers in [14] investigated the polytropic sort, which is important in Newtonian astrophysics, and used the Elzaki transform homotopy perturbation approach (ETHPT) in their strategy to solve the nonlinear Emden–Fowler model. They also used the homotopy disturbance approach to manage the nonlinear term with ease. For the purpose of modeling a nonlinear biological reaction model, the Laplace transform method was utilized in [15] to provide an approximate solution. They asserted that the applicable method, which can be used to solve any linear or nonlinear differential equation, is the most intriguing and straightforward approach that yields remarkably accurate results. In [16], nonlinear Rubella Sickness Tributes are unwound using a numerical approach. The SVLPT's quality is demonstrated. The limited contrast system is used to understand the condition's game strategy. The mathematical model is provided to attest to the steadfast quality and sufficiency of the specified technique. Advancing research, the numerical solution and dynamical behaviors of a fractional nonlinear Rubella disease model were studied in [17]. They proved the existence and stability of the solution of the fractional order model. The optimal control of the model and the numerical technique for the simulation of the control problem were also discussed.

Fractional calculus is a branch of mathematics that studies the properties of non-integer order integrals and derivatives (also known as fractional derivatives and integrals, or fractional integrals and fractional derivatives, respectively). This topic focuses on the concept of and strategies for solving differential equations with fractional derivatives of an unknown function (called fractional differential equations). The origins of fractional differential equations may be traced back to the invention of classical calculus in a letter Leibniz wrote to L'Hospital in the year 1695, when the notion of a semi-derivative was proposed.

Researchers in fractional calculus look at the characteristics of non-integer-order derivatives and integrals. Numerous applications from various researchers have been extensively reviewed. In [18], a fractional differential system in a restricted domain of the classical control theory approach for a differential system with a time delay was investigated. It takes into consideration the FOCP, or fractional optimum control problem. This same fractional optimal control was extended by [19] for problems involving variable-order differential systems. In research presented in [20], a time-fractional model of HIV-I infection of CD4^+^ T lymphocyte cells in an uncertain environment was studied. First, the fractional fuzzy HIV-I model is converted to an interval-based fuzzy differential equation by using the single parametric form, and they applied the fractional differential transform method to solve the corresponding fractional model, obtaining the required solution in terms of intervals. The analysis of the dynamics of phytoplankton nutrient and whooping cough models with a nonsingular kernel arising in the biological system was studied in [21]. The Atangana–Baleanu derivative was used in the fractional-order model to examine the phytoplankton nutrient and whooping cough models. The existence and uniqueness of the solution of these models were shown using the fixed point theorem, and the solutions for the fractional-order models are obtained using the perturbation transform method, namely HPETM. Detailed simulations are carried out to show the effect of fractional order. Investigation of a new modeling and existence-uniqueness analysis for Babesiosis disease was carried out in [22], advancing in the study and application of fractional calculus. A fractional epidemiology model of the disease and processes of Babesiosis transmission specified in the fractional derivative sense is used to model and analyze the transmission of this parasite, treat this parasite, and deal with this essential issue. The propagation of Babesiosis mechanisms is studied using the Caputo–Fabrizio (CF) derivative. Also, a SIR epidemic model of childhood diseases through fractional operators with Mittag–Leffler and exponential kernels was studied in [23]. They applied the homotopy perturbation Elzaki transform method to obtain the solutions of the epidemic model of childhood diseases involving time-fractional order Atangana–Baleanu and Caputo–Fabrizio derivatives and Their study revealed that the method offers a rapidly convergent series solution. A research project presented in [24] on the nonlinear fractional tumor-immune model is solved using the reduced differential transform technique. In the Caputo sense, the fractional derivative is described. This approach yields straightforward and very precise solutions. The signal flow diagram of the model reveals the model. Additionally, a simulation of the system using MATLAB's Simulink is provided.

In the studies presented in [25] for variational inequalities, the problem of fractional optimal control is taken into account. The fractional time derivative is considered in the Riemann–Liouville definition. The existence and uniqueness of a class of fractional differential variational inequalities are investigated in a Sobolev space. For a fractional variational inequality in a constrained domain with Dirichlet and Neumann boundary conditions, a solution is demonstrated. Controls have restrictions imposed on them. For the quadratic performance functional in the fractional Cauchy problem, we construct the required and sufficient optimality requirements. Examples include the description of the adjoint issue and the first-order optimality criterion for the Euler–Lagrange equation.

In order to stop the COVID-19 pandemic, control methods relating to health care that may be implemented, such as isolation, quarantine, and travel restrictions, were presented in [6]. To better understand the dynamical behavior of nonlinear COVID-19 models, two techniques, namely the homotopy perturbation method (HPM) and the modified reduced differential transform approach, were used. According to their findings, social exclusion of those who may be afflicted is a powerful tool for halting the spread of the fatal virus. In [26], a new Corona virus mathematical model and dynamics are presented, and a brief description of the interactions between bats and unidentified hosts, then between people and the infection reservoir (the seafood market), is given. The formulated model is then used to create a fractional model that is numerically solved and produces graphical results that can help reduce infection.

As a motivation for this study, there is a need for a dependable strategic procedure capable of containing the spread of the COVID-19 virus in Nigeria to reduce the pressure exerted by the first wave of the COVID-19 pandemic on the country’s struggling health care system because of the risk posed by the second wave of the COVID-19 virus. We modified a mathematical model presented in [27] by formulating its derivative to be of Caputo fractional order and adding a vaccinated compartment in order to analyze the effect of the fractional order derivative and vaccination of individuals in tracking the spread of COVID-19 transmission. Following that, we demonstrate the model's fundamental characteristics, which are the validity of its existence and uniqueness, and establish its basic reproduction number. Finally, LADM was used to obtain the analytical solution, and we examined the effect of the Caputo fractional order derivative on its classes and parameters using the Laplace-Adomian decomposition method.

## Preliminaries

### Fractional calculus

Here, some fundamental definitions and properties in fractional calculus applicable in this paper will be given.

#### **Definition 1**

A real function $\rho (x),\,\,x > 0,$ is said to be in the space $C_{\mu \,} ,\,\,\mu \in R$ if there exist a real number $m > \mu$ such that $\rho (x) = x^{m} \rho_{1} (x).$ where $\rho_{1} (x) \in C(0,\infty ),$ and it is said to be in the space $C^{n}_{\mu \,}$ if and only if $\rho^{(n)} \in C_{\mu } ,\,n \in N.$

#### **Definition 2**

The Riemann–Liouville fractional integration of order $\eta \ge 0$ of a positive real function $\rho (x) \in C_{\mu \,} ,\,\,\mu \ge - 1\,\,\,x > 0$ is defined as:

$$I^{\eta } \rho (x) = \frac{1}{\Gamma (\eta )}\int\limits_{0}^{t} {(x - t)^{\eta - 1} \rho (x){\text{d}}x}$$ Such that $$I^{0} \rho (x) = \rho (x).$$

The following properties hold for fractional integral operator $I^{\eta }$ for $\rho (x) \in C_{\mu \,} ,\,\,\mu \ge - 1\,\,\,\eta ,\alpha \ge 0\,$ and $\,\beta \ge - 1$:

$$\begin{aligned} & 1.\,\,\,\,I^{\eta } I^{\alpha } \rho (x) = I^{\eta + \alpha } \rho (x), \\ & 2.\,\,\,I^{\eta } I^{\alpha } \rho (x) = I^{\alpha } I^{\beta } \rho (x), \\ & 3.\,\,I^{\eta } x^{\beta } = \frac{\Gamma (\beta + 1)}{{\Gamma (\eta + \beta + 1)}}x^{\eta + \beta } . \\ \end{aligned}$$

#### **Definition 3**

The Caputo fractional derivative of a positive real function $\rho (x)$ given as $D^{\eta } \rho (x)$ is given by.

$$D^{\eta } \rho (x) = \frac{1}{\Gamma (n - 1)}\int\limits_{0}^{t} {(x - t)^{n - \beta - 1} \rho^{(n)} (t){\text{d}}t}$$ For $$n - 1 < \eta \le n,\,\,n \in N,\,\,\,t > 0,\,\,\varphi \in \mathop c\nolimits_{ - 1}^{n} .$$

The following property holds for fractional integration of the Caputo fractional derivative. For $n - 1 < \eta \le n,\,\,n \in N,\,\,\varphi \in \mathop c\nolimits_{ - 1}^{n} ,\,\mu \ge - 1.$ Then

$$I^{\eta } D^{\eta } \rho (x) = \rho (x) - \sum\limits_{k = 0}^{n - 1} {\rho^{(k)} (0)\frac{{x^{k} }}{k!}}$$

### Laplace transform

In this section, we give some basic properties of the Laplace transform method as applied in the article.

#### **Definition 4**

Let $\varphi (t)$ be a function defined for all positive real number $t \ge 0$.

i. The Laplace transform of $\varphi (t)$ is the function $\varphi (s):\varphi (s) = \int\limits_{0}^{\infty } {e^{ - st} \varphi (t)} {\text{d}}t$
ii. The Laplace transform of function $\varphi (t)$ with order η is defined as $L[\varphi^{\eta } (t)] = \alpha^{\eta } L[\varphi (t)] - \alpha^{\eta - 1} \varphi (0) - \alpha^{\eta - 2} \varphi \prime (0) - \alpha^{\eta - 3} \varphi \prime \prime (0) \cdots$
iii. The inverse Laplace transform of $\frac{\varphi (s)}{s}$ is $L^{ - 1} \frac{\varphi (s)}{s} = \int\limits_{0}^{t} {\varphi (t){\text{d}}t}$

#### **Definition 5**

The Laplace transform of the fractional integral and derivatives for $\alpha > 0$ is defined as: $L[D_{t}^{\alpha } \rho (x)] = L\left[ {\frac{1}{\Gamma (n - \alpha )}\frac{{{\text{d}}^{n} }}{{{\text{d}}x^{n} }}\int_{0}^{t} {(x - u)^{n - \alpha - 1} \rho (x){\text{d}}x} } \right]$.

### Adomian decomposition method

#### **Definition 6**

The Adomian polynomials denoted by $A_{0} ,A_{1} , \ldots A_{n}$, consists in the decomposition the unknown function $y(t)$ in a series of the form $y(t) = y_{0} + y_{1} + y_{2} + \cdots y_{n}$ can be expressed as:

$$A_{n} = \frac{1}{n}\frac{{{\text{d}}^{n} }}{{{\text{d}}\lambda^{n} }}\left[ {G(t)\sum\limits_{j = 0}^{n} {y_{j} \lambda^{j} } } \right]_{\lambda = 0}$$

### Existence and uniqueness of solution

#### **Theorem 1**

Let

$$\begin{aligned} & x_{1}^{^{\prime}} = f_{1} (x_{1} ,x_{2} ,x_{3} , \ldots x_{n} ,t),x_{1} (t_{0} ) = x_{1} 0 \\ & x^{^{\prime}}_{2} = f_{2} (x_{1} ,x_{2} ,x_{3} , \ldots x_{n} ,t),x_{2} (t_{0} ) = x_{2} 0 \\ & x^{^{\prime}}_{3} = f_{3} (x_{1} ,x_{2} ,x_{3} , \ldots x_{n} ,t),x_{2} (t_{0} ) = x_{3} 0 \\ & \vdots \\ & x^{^{\prime}}_{n} = f_{n} (x_{1} ,x_{2} ,x_{3} , \ldots x_{n} ,t),x_{n}^{{}} (t_{0} ) = x_{n} 0 \\ \end{aligned}$$

*Suppose D is the region in* (*n* + 1) *dimensional space (one dimension) for* $t$ *and* $n$ *dimension for the vector x, if the partial derivative* $\frac{{{\text{d}}f}}{{{\text{d}}x_{i} }}$ *where* $i = 1,2,3, \ldots n$ *are continuous in* $D = \{ (x,t):\backslash t - t_{0} \backslash \le a,\backslash x - x_{0} \backslash \le b$ *then there constant* $\delta \ge 0$ *Such that there exist a unique*.

*Continuous vector* $x = [x_{1} (t),x_{2} (t),x_{3} (t), \ldots x_{n} (t)]$ *solution in the interval* $\backslash t - t_{0} \backslash \le \delta$.

### Stability analysis

#### **Lemma 1**

*Suppose* $\overline{x}$ *is an equilibrium point of a time-invariant system* i.e., $\overline{x}$ *is an equilibrium point of* $X(t) = f(X(t))$.

*For a precise definition of stability, it is convenient to introduce the notation* $S(\overline{X},R)$ *to denote a spherical region in the state space with center at* $\overline{x}$ *and Radius R*.

#### **Definition 5**

An equilibrium point $\overline{x}$ is stable if there exists $R_{0} > 0$ for which the following is true. For every $R < R_{0}$ there exist *r*. $0 < r < R$,such that $X(0)$ is inside $S(\overline{X},R)$,when $X(t)$ is inside $S(\overline{X},R)$ for all $t > 0$.

#### **Definition 6**

An equilibrium point $\overline{x}$ unstable if it is not stable. Equivalently, $\overline{x}$ unstable $R_{0} > 0$ for which the following is true. For every $R < R_{0}$ there exist r. $0 < r < R$, such that $X(0)$ is inside $S(\overline{X},R)$,when $X(t)$ is inside $S(\overline{X},R)$ for all $t > 0$.

## Methods

The suggested model's composition is described in this section. A nonlinear mathematical model is constructed and tested to investigate the influence of transmission coefficient and other epidemiological factors on the dynamic transmission of COVID-19 virus. Consider the deterministic mathematical model employing the Corona virus illness model to have a better grasp of the Corona virus disease model.

The total population is given as:

1 $$N(t) = S(t) + V(t) + E(t) + I(t) + P(t) + A(t) + H(t) + R(t) + F(t).$$

A vulnerable population member is someone who is at risk of developing a disease. Owing to the recruitment of active persons or the birth of individuals, the proportion of susceptible rises at rate rho, whilst the population falls due to natural mortality.

2 $$K_{1} I(t)S(t) + K_{2} H(t)S(t) + K_{3} P(t)S(t).$$

if the infection's propelling force, then the population's rate of change is

determined by

3 $$\frac{{{\text{d}}S(t)}}{{{\text{d}}t}} = \pi (1 - \rho ) - K_{1} I(t)S(t) + K_{2} H(t)S(t) + K_{3} P(t)S(t) + \omega V(t\,).$$

It calculates coefficient of individual human-to-human transmission.

People who have received the COVID-19 vaccination are included in the Vaccinated population. The population of vaccinated people grows with the rate of recruiting vaccinated people and decreases with the rate of vaccination death, where is the rate of vaccination loss.

Rate of change of vaccinated population is thus given by:

4 $$\frac{{{\text{d}}V(t)}}{{{\text{d}}t}} = \pi \rho - (\omega + \delta_{v} )V(t).$$

The population of Exposed (*E*) individual is a member of a population generated through infection of susceptible that infected but not yet infectious, exposed individual increase through the infection of susceptible by the force of infection $K_{1} I(t)S(t) + K_{2} H(t)S(t) + K_{3} P(t)S(t)$ and decrease individual leaves the exposed class at $\kappa$.

The, changing rate of the vaccinated individual is given by:

5 $$\frac{{{\text{d}}E(t)}}{{{\text{d}}t}} = K_{1} I(t)S(t) + K_{2} H(t)S(t) + K_{3} P(t)S(t) - \kappa E(t).$$

A person who displays symptoms of the COVID-19 virus is deemed symptomatic and infectious, and their chance of catching the virus from another person increases as a result of exposure. Both natural mortality and disease-related mortality caused by infected persons have a detrimental influence on this population.

6 $$\frac{{{\text{d}}I(t)}}{{{\text{d}}t}} = \kappa \rho_{1} E(t) - \gamma_{a} I(t) - \gamma_{1} I(t) - \delta_{1} I(t\,).$$

A Super-Spreader is a member of the population who has the virus and is more likely to distribute it to others. As more people are exposed to the virus through human-to-human transmission, the population rises. Individuals are also being reduced due to natural death rates and disease-related mortality rates caused by sick persons.

7 $$\frac{{{\text{d}}P(t)}}{{{\text{d}}t}} = \kappa \rho_{2} E(t) - (\gamma_{a} + \gamma_{1} )P(t) - \delta_{1} P(t).$$

The population of Infectious but Asymptomatic individuals is a member of a population that does not show symptoms of COVID-19 virus at all, They increased by the infection progression at a rate $(1 - \rho_{1} - \rho_{2} )$ which causes the development of symptoms by exposed individual $(\kappa )$ becoming asymptomatic, This population decreases as a result of induced deaths from disease and natural death due to infected individual $(\delta_{i} )$, Hence

8 $$\frac{{{\text{d}}A(t)}}{{{\text{d}}t}} = \kappa (1 - \rho_{1} - \rho_{2} )E(t).$$

The population of the Hospitalized individual is a member of the population that show symptoms of COVID-19 Virus, symptomatic and super-spreader individual becomes hospitalized at rate $(\gamma_{a} )$. This population increase by the individual exposed to the virus that is human-to human transmission of symptomatic and super-spreader individual and also decreased by natural and disease-induced death rate due to infected individual $(\delta_{i} )$, Hence

9 $$\frac{{{\text{d}}H(t)}}{{{\text{d}}t}} = I(t)\gamma_{a} + P(t)\gamma_{a} - \gamma_{r} H(t) - \delta_{h} H(t).$$

The population of recovered individuals is a member of the population who recovered from the disease; this population rose at the rate of recovery without being hospitalized and decreased as a consequence of the efficacy rate of recovering in hospitalized patients.

10 $$\left. {\begin{array}{*{20}l} {\frac{{{\text{d}}S(t)}}{{{\text{d}}t}} = \pi (1 - \rho ) - K_{1} I(t)S(t) + K_{2} H(t)S(t) + K_{3} P(t)S(t) + \omega V(t),} \hfill \\ {\frac{{{\text{d}}V(t)}}{{{\text{d}}t}} = \pi \rho - (\omega + \delta_{v} )V(t),} \hfill \\ {\frac{{{\text{d}}E(t)}}{{{\text{d}}t}} = K_{1} I(t)S(t) + K_{2} H(t)S(t) + K_{3} P(t)S(t) - \kappa E(t),} \hfill \\ {\frac{{{\text{d}}I(t)}}{{{\text{d}}t}} = \kappa \rho_{1} E(t) - \gamma_{a} I(t) - \gamma_{1} I(t) - \delta_{1} I(t),} \hfill \\ {\frac{{{\text{d}}P(t)}}{{{\text{d}}t}} = \kappa \rho_{2} E(t) - (\gamma_{a} + \gamma_{1} )P(t) - \delta_{1} P(t),} \hfill \\ {\frac{{{\text{d}}A(t)}}{{{\text{d}}t}} = \kappa (1 - \rho_{1} - \rho_{2} )E(t),} \hfill \\ {\frac{{{\text{d}}H(t)}}{{{\text{d}}t}} = I(t)\gamma_{a} + P(t)\gamma_{a} - H(t)\gamma_{r} - H(t)\delta_{h} ,} \hfill \\ {\frac{{{\text{d}}R(t)}}{{{\text{d}}t}} = \gamma_{1} I(t) + \gamma_{1} P(t) - H(t)\gamma_{r} ,} \hfill \\ {\frac{{{\text{d}}F(t)}}{{{\text{d}}t}} = \delta_{1} I(t) + \delta_{p} P(t) + \delta_{h} H(t) + \delta_{v} V(t).} \hfill \\ \end{array} } \right\}$$

where $K_{1} = \frac{\beta }{N}$, $K_{2} = \frac{l\beta }{N}$ and $K_{3} = \frac{{\beta^{^{\prime}} }}{N}$ (Table 1).

**Table 1:** Variables and parameters descriptions

$S(t)$ Susceptible population at given time *t*
$V(t)$ Vaccinated population at given time *t*
$E(t)$ Those that were exposed at given time *t*
$I(t)$ Symptomatic and infectious individuals in the population at given time *t*
$P(t)$ Super-spreader population at time given *t*
$A(t)$ The infected but asymptomatic population at time *t*
$H(t)$ Hospitalized population at time *t*
$R(t)$ Recovering population at time *t*
$F(t)$ Dead or fatality class population
$$\pi$$ Recruitment rate
$K_{1} I(t)S(t) + K_{2} H(t)S(t) + K_{3} P(t)S(t)$ is force of infection
$\beta$ Measures human-to-human transmission coefficient
$\beta^{^{\prime}}$ Measures transmission coefficient of a super-spreader
$l$ measures the relative transmission risk of hospitalized individuals
$\kappa$ the rate in which people leave the exposed class
$\rho$ The rate at of recruiting the vaccinated individual
$\omega$ The rate of losing the vaccination
$\delta_{v}$ The death rate due to the vaccination
$\rho_{1}$ fraction of change from exposed class to symptomatic
$\rho_{2}$ Comparatively less people become super-spreaders when they are exposed
$1 - \rho_{1} - \rho_{2}$ from the exposed to the asymptomatic class
$\gamma_{a}$ the standard hospitalization rate for symptomatic super-spreader individuals
$\gamma_{1}$, $\gamma_{r}$ Hospitalized recovery rate
$\delta_{i}$, $\delta_{p}$, $\delta_{h}$ Infected, super-spreader, and hospitalized populations' relative disease-related death rates

### Existence and uniqueness of solution

Theorem 1: Let

$$\begin{aligned} & z_{1}^{^{\prime}} = g_{1} (z_{1} ,z_{2} ,z_{3} , \ldots z_{n} ,t),z_{1} (t_{0} ) = z_{10} \\ & z^{^{\prime}}_{2} = g_{2} (z_{1} ,z_{2} ,z_{3} , \ldots z_{n} ,t),z_{2} (t_{0} ) = z_{20} \\ & z^{^{\prime}}_{3} = g_{3} (z_{1} ,z_{2} ,z_{3} , \ldots z_{n} ,t),z_{2} (t_{0} ) = z_{30} \\ & \vdots \\ & z^{^{\prime}}_{n} = g_{n} (z_{1} ,z_{2} ,z_{3} , \ldots x_{n} ,t),z_{n}^{{}} (t_{0} ) = z_{n0} \\ \end{aligned}$$

Let *R* be the vector’s area dimensional space (one dimension), if the partial derivative.

$\frac{{{\text{d}}g}}{{{\text{d}}z_{i} }}$ where $i = 1,2,3 \ldots n$ are continuous in $\Re = \{ (z,t):\left| {t - t_{0} } \right| \le a,\left| {z - z_{0} } \right| \le b$ then there constant $\delta \ge 0$ Such that there exists a unique continuous vector solution $z = [z_{1} (t),z_{2} (t),z_{3} (t), \ldots z_{n} (t)]$ in the interval $\left| {t - t_{0} } \right| \le \delta$.

Let:

11 $$\begin{aligned} g_{1} & = \frac{{{\text{d}}S}}{{{\text{d}}t}} = \pi (1 - \rho ) - \, K_{1} I(t)S(t) + K_{2} H(t)S(t) + K_{3} P(t)S(t) \, + \omega V(t), \\ g_{2} & = \frac{{{\text{d}}V}}{{{\text{d}}t}} = \pi \rho - (\omega + \delta_{v} )V(t), \\ g_{3} & = \frac{{{\text{d}}E}}{{{\text{d}}t}} = K_{1} I(t)S(t) + K_{2} H(t)S(t) + K_{3} P(t)S(t) - \kappa E(t), \\ g_{4} & = \frac{{{\text{d}}I}}{{{\text{d}}t}} = \kappa \rho_{1} E(t) - \gamma_{a} I(t) - \gamma_{1} I(t) - \delta_{1} I(t), \\ g_{5} & = \frac{{{\text{d}}P}}{{{\text{d}}t}} = \kappa \rho_{2} E(t) - (\gamma_{a} + \gamma_{1} )P(t) - \delta_{1} P(t), \\ g_{6} & = \frac{{{\text{d}}A}}{{{\text{d}}t}} = \kappa (1 - \rho_{1} - \rho_{2} )E(t), \\ g_{7} & = \frac{{{\text{d}}H}}{{{\text{d}}t}} = I(t)\gamma_{a} + P(t)\gamma_{a} - \gamma_{r} H(t) - \delta_{h} H(t), \\ g_{8} & = \frac{{{\text{d}}R}}{{{\text{d}}t}} = \gamma_{1} I(t) + \gamma_{1} P(t) - \gamma_{r} H(t), \\ g_{9} & = \frac{{{\text{d}}F}}{{{\text{d}}t}} = \delta_{1} I(t) + \delta_{p} P(t) + \delta_{h} H(t) + \delta_{v} V(t). \\ \end{aligned}$$

$$\Re = \left\{ \begin{gathered} \left( {S(t),V(t),E(t),I(t),P(t),A(t),H(t),R(t),F(t)} \right):\left| {S - S_{0} } \right| \le a, \hfill \\ \left| {V - V_{0} } \right| \le b,\left| {E - E_{0} } \right| \le c,\left| {I - I_{0} } \right| \le d,\left| {P - P_{0} } \right| \le e, \hfill \\ \left| {A - A_{0} } \right| \le f,\left| {H - H_{0} } \right| \le g,\left| {R - R_{0} } \right| \le h,\left| {F - F_{0} } \right| \le i \hfill \\ \end{gathered} \right\}$$

The solution to the model is unique, taking partial derivative to obtain the following;

$$g_{1} = \frac{{{\text{d}}S}}{{{\text{d}}t}} = \pi (1 - \rho ) - K_{1} I(t)S(t) + K_{2} H(t)S(t) + K_{3} P(t)S(t) + \omega V(t).$$

$$\left| {\frac{{\partial g_{1} }}{\partial S}} \right| = 0,\left| {\frac{{\partial g_{1} }}{\partial V}} \right| = \left| \omega \right|,\left| {\frac{{\partial g_{1} }}{\partial E}} \right| = 0,\left| {\frac{{\partial g_{1} }}{\partial I}} \right| = \left| { - \beta } \right|,\left| {\frac{{\partial g_{1} }}{\partial P}} \right| = \left| { - \beta^{^{\prime}} } \right|,\left| {\frac{{\partial g_{1} }}{\partial A}} \right| = 0,\left| {\frac{{\partial g_{1} }}{\partial H}} \right| = \left| { - l\beta } \right|,\left| {\frac{{\partial g_{1} }}{\partial R}} \right| = 0,\left| {\frac{{\partial g_{1} }}{\partial F}} \right| = 0$$

Also, taking partial derivative of the second function i.e.,

$g_{2} = \frac{{{\text{d}}V}}{{{\text{d}}t}} = \pi \rho - (\omega + \delta_{v} )V(t)$ to obtain the following;

$$\left| {\frac{{\partial g_{2} }}{\partial S}} \right| = 0,\left| {\frac{{\partial g_{2} }}{\partial V}} \right| = \left| { - (\omega + \delta_{v} )} \right|,\left| {\frac{{\partial g_{2} }}{\partial E}} \right| = 0,\left| {\frac{{\partial g_{2} }}{\partial I}} \right| = 0,\left| {\frac{{\partial g_{2} }}{\partial P}} \right| = 0,\left| {\frac{{\partial g_{2} }}{\partial A}} \right| = 0,\left| {\frac{{\partial g_{2} }}{\partial H}} \right| = 0,\left| {\frac{{\partial g_{2} }}{\partial R}} \right| = 0,\left| {\frac{{\partial g_{2} }}{\partial R}} \right| = 0$$

Similarly, taking the partial derivative of the third function i.e., $g_{3} = \frac{{{\text{d}}E}}{{{\text{d}}t}} = K_{1} I(t)S(t) + K_{2} H(t)S(t) + K_{3} P(t)S(t) - \kappa E(t)$,

to obtain the following;

$\left| {\frac{{\partial g_{3} }}{\partial S}} \right| = 0,\left| {\frac{{\partial g_{3} }}{\partial V}} \right| = \left| { - (\omega + \delta_{v} )} \right|,\left| {\frac{{\partial g_{3} }}{\partial E}} \right| = \left| { - \kappa } \right|,\left| {\frac{{\partial g_{3} }}{\partial I}} \right| = \beta ,\left| {\frac{{\partial g_{3} }}{\partial P}} \right| = \left| {\beta^{^{\prime}} } \right|,\left| {\frac{{\partial g_{3} }}{\partial A}} \right| = 0,\left| {\frac{{\partial g_{3} }}{\partial H}} \right| = \left| {l\beta } \right|,\left| {\frac{{\partial g_{3} }}{\partial R}} \right| = 0,\left| {\frac{{\partial g_{3} }}{\partial F}} \right| = 0$ Similarly, taking the partial derivative of fourth function i.e.,

$g_{4} = \frac{{{\text{d}}I}}{{{\text{d}}t}} = \kappa \rho_{1} E(t) - \gamma_{a} I(t) + \gamma_{1} I(t) - \delta_{1} I(t)$ to obtain the following;

$$\left| {\frac{{\partial g_{4} }}{\partial S}} \right| = 0,\left| {\frac{{\partial g_{4} }}{\partial V}} \right| = 0,\left| {\frac{{\partial g_{4} }}{\partial E}} \right| = \left| {\kappa \rho_{1} } \right|,\left| {\frac{{\partial g_{4} }}{\partial I}} \right| = \left| { - (\gamma_{a} + \gamma_{1} ) - \delta_{1} } \right|,\left| {\frac{{\partial g_{4} }}{\partial P}} \right| = 0,\left| {\frac{{\partial g_{4} }}{\partial A}} \right| = 0,\left| {\frac{{\partial g_{4} }}{\partial H}} \right| = 0,\left| {\frac{{\partial g_{4} }}{\partial R}} \right| = 0,\left| {\frac{{\partial g_{4} }}{\partial R}} \right| = 0$$

Taking the partial derivative of the fifth function i.e., $g_{5} = \frac{{{\text{d}}P}}{{{\text{d}}t}} = \kappa \rho_{2} E(t) - \gamma_{a} P(t) - \gamma_{1} P(t) - \delta_{1} P(t)$ to obtain the following;

$$\begin{aligned} & \left| {\frac{{\partial g_{6} }}{\partial S}} \right| = 0,\left| {\frac{{\partial g_{6} }}{\partial V}} \right| = 0,\left| {\frac{{\partial g_{6} }}{\partial E}} \right| = \left| {\kappa (1 - \rho_{1 - } \rho_{2} )} \right|,\left| {\frac{{\partial g_{6} }}{\partial I}} \right| = 0,\left| {\frac{{\partial g_{6} }}{\partial P}} \right| = 0,\left| {\frac{{\partial g_{6} }}{\partial A}} \right| = 0,\left| {\frac{{\partial g_{6} }}{\partial H}} \right| = 0,\left| {\frac{{\partial g_{6} }}{\partial R}} \right| = 0,\left| {\frac{{\partial g_{6} }}{\partial F}} \right| = 0 \\ & \left| {\frac{{\partial g_{5} }}{\partial S}} \right| = 0,\left| {\frac{{\partial g_{5} }}{\partial V}} \right| = 0,\left| {\frac{{\partial g_{5} }}{\partial E}} \right| = \left| {\kappa \rho_{2} } \right|,\left| {\frac{{\partial g_{5} }}{\partial I}} \right| = 0,\left| {\frac{{\partial g_{5} }}{\partial P}} \right| = \left| { - (\gamma_{a} + \gamma_{1} ) - \delta_{1} } \right|,\left| {\frac{{\partial g_{5} }}{\partial A}} \right| = 0,\left| {\frac{{\partial g_{5} }}{\partial H}} \right| = 0,\left| {\frac{{\partial g_{5} }}{\partial R}} \right| = 0,\left| {\frac{{\partial g_{5} }}{\partial F}} \right| = 0 \\ \end{aligned}$$ Taking the partial derivative of sixth function i.e., $g_{6} = \frac{{{\text{d}}A}}{{{\text{d}}t}} = \kappa (1 - \rho_{1} - \rho_{2} )E(t)$ yield the following;

Taking the partial derivative of seventh function i.e., $g_{7} = \frac{{{\text{d}}H}}{{{\text{d}}t}} = \gamma_{a} I(t) + \gamma_{a} P(t) - \gamma_{r} H(t) - \delta_{h} H(t)$ yield the following;

$$\left| {\frac{{\partial g_{7} }}{\partial S}} \right| = 0,\left| {\frac{{\partial g_{7} }}{\partial V}} \right| = 0,\left| {\frac{{\partial g_{7} }}{\partial E}} \right| = 0,\left| {\frac{{\partial g_{7} }}{\partial I}} \right| = \left| {\gamma_{a} } \right|,\left| {\frac{{\partial g_{7} }}{\partial P}} \right| = \left| {\gamma_{a} } \right|,\left| {\frac{{\partial g_{7} }}{\partial A}} \right| = 0,\left| {\frac{{\partial g_{7} }}{\partial H}} \right| = \left| { - \gamma_{a} - \delta_{h} } \right|,\left| {\frac{{\partial g_{7} }}{\partial R}} \right| = 0,\left| {\frac{{\partial g_{7} }}{\partial F}} \right| = 0$$

Taking partial derivative of eighth function i.e.,

$$g_{8} = \frac{{{\text{d}}R}}{{{\text{d}}t}} = \gamma_{1} (I(t) + P(t)) - \gamma_{r} H(t)$$ yield following;

$$\left| {\frac{{\partial g_{8} }}{\partial S}} \right| = 0,\left| {\frac{{\partial g_{8} }}{\partial V}} \right| = 0,\left| {\frac{{\partial g_{8} }}{\partial E}} \right| = 0,\left| {\frac{{\partial g_{8} }}{\partial I}} \right| = \left| {\gamma_{1} } \right|,\left| {\frac{{\partial g_{8} }}{\partial P}} \right| = \left| {\gamma_{1} } \right|,\left| {\frac{{\partial g_{8} }}{\partial A}} \right| = 0,\left| {\frac{{\partial g_{8} }}{\partial H}} \right| = \left| { - \gamma_{r} } \right|,\left| {\frac{{\partial g_{8} }}{\partial R}} \right| = 0,\left| {\frac{{\partial g_{8} }}{\partial F}} \right| = 0$$

Finally, taking the partial derivative of ninth function.

i.e., $g_{9} = \frac{{{\text{d}}F}}{{{\text{d}}t}} = \delta_{1} I(t) + \delta_{p} P(t) + \delta_{h} H(t) + \delta_{v} V(t)$, yield the following;$\left| {\frac{{\partial g_{8} }}{\partial F}} \right| = 0,\left| {\frac{{\partial g_{9} }}{\partial V}} \right| = \left| {\delta_{v} } \right|,\left| {\frac{{\partial g_{9} }}{\partial E}} \right| = 0,\left| {\frac{{\partial g_{9} }}{\partial I}} \right| = \left| {\delta_{1} } \right|,\left| {\frac{{\partial g_{9} }}{\partial P}} \right| = \left| {\delta_{p} } \right|,\left| {\frac{{\partial g_{9} }}{\partial A}} \right| = 0,\left| {\frac{{\partial g_{9} }}{\partial H}} \right| = \left| {\delta_{h} } \right|,\left| {\frac{{\partial g_{9} }}{\partial R}} \right| = 0,\left| {\frac{{\partial g_{9} }}{\partial F}} \right| = 0$.

Because the partial derivative exists, it is continuous and bounded, as shown in Theorem 1.

The problem has a unique solution, showing that it is mathematically and biologically properly stated.

### Basic reproduction number

In mathematical epidemiology, one of the core issues is to establish the threshold condition that governs whether or not an infectious disease may spread in a susceptible population which is defined as basic reproduction number.

Let,

$$F = \left( {\begin{array}{*{20}c} {\begin{array}{*{20}c} 0 \\ 0 \\ 0 \\ 0 \\ \end{array} } & {\begin{array}{*{20}c} \beta \\ 0 \\ 0 \\ 0 \\ \end{array} } & {\begin{array}{*{20}c} {l\beta } \\ 0 \\ 0 \\ 0 \\ \end{array} } & {\begin{array}{*{20}c} {\beta^{^{\prime}} } \\ 0 \\ 0 \\ 0 \\ \end{array} } \\ \end{array} } \right),V = \left( {\begin{array}{*{20}c} {\begin{array}{*{20}c} \kappa \\ { - \kappa \rho_{1} } \\ { - \kappa \rho_{2} } \\ 0 \\ \end{array} } & {\begin{array}{*{20}c} 0 \\ {(\gamma_{a} + \gamma_{1} ) + \delta_{1} } \\ 0 \\ {\gamma_{a} } \\ \end{array} } & {\begin{array}{*{20}c} 0 \\ 0 \\ 0 \\ {\gamma_{1} + \delta_{h} } \\ \end{array} } & {\begin{array}{*{20}c} 0 \\ 0 \\ {(\gamma_{a} + \gamma_{1} ) + \delta_{1} } \\ {\gamma_{1} } \\ \end{array} } \\ \end{array} } \right)$$

$$R_{0} = \frac{{l\beta (\kappa \rho_{1} )(\gamma_{a} + \gamma_{1} + \delta_{p} )\gamma_{a} + ((\gamma_{a} + \gamma_{1} + \delta_{p} )(\kappa \rho_{1} )(\gamma_{r} + \delta_{p} ) + \beta (\kappa \rho_{2} )(\gamma_{a} + \gamma_{1} ) + \delta_{1} )(\gamma_{r} + \delta_{h} ) + \beta l(\kappa \rho_{2} )(\gamma_{a} + \gamma_{1} ) + \delta_{1} )}}{{\kappa (\gamma_{a} + \gamma_{1} + \delta_{p} )(\gamma_{a} + \delta_{h} )(\gamma_{a} + \gamma_{1} + \delta_{p} )}}$$

### Application of Laplace Adomian decomposition method

We derive the Caputo fractional-order derivative by substituting it for the derivative in the mathematical model for fractional-order of coronavirus disease shown in (10), so we obtain (12)

12 $$\left. {\begin{array}{*{20}l} {{}^{c}D^{{\alpha_{1} }} S(t) = \pi (1 - \rho ) - K_{1} I(t)S(t) + K_{2} H(t)S(t) + K_{3} P(t)S(t) + \omega V(t),} \hfill \\ {{}^{c}D^{{\alpha_{2} }} V(t) = \pi \rho - (\omega + \delta_{v} )V(t),} \hfill \\ {{}^{c}D^{{\alpha_{3} }} E(t) = K_{1} I(t)S(t) + K_{2} H(t)S(t) + K_{3} P(t)S(t) - \kappa E(t),} \hfill \\ {{}^{c}D^{{\alpha_{4} }} I(t) = \kappa \rho_{1} E(t) - (\gamma_{a} + \gamma_{1} )I(t) - \delta_{1} I(t),} \hfill \\ {{}^{c}D^{{\alpha_{5} }} P(t) = \kappa \rho_{2} E(t) - (\gamma_{a} + \gamma_{1} )P(t) - \delta_{1} P(t),} \hfill \\ {{}^{c}D^{{\alpha_{6} }} A(t) = \kappa (1 - \rho_{1} - \rho_{2} )E(t),} \hfill \\ {{}^{c}D^{{\alpha_{7} }} H(t) = \gamma_{a} I(t) + \gamma_{a} P(t) - \gamma_{r} H(t) - \delta_{h} H(t),} \hfill \\ {{}^{c}D^{{\alpha_{8} }} R(t) = \gamma_{1} I(t) + \gamma_{1} P(t) - \gamma_{r} H(t),} \hfill \\ {{}^{c}D^{{\alpha_{9} }} F(t) = \delta_{1} I(t) + \delta_{p} P(t) + \delta_{h} H(t) + \delta_{v} V(t).} \hfill \\ \end{array} } \right\}$$

Initial condition $$S_{0} = n_{1} ,V_{0} = n_{2,} ,E_{0} = n_{3} ,I_{0} = n_{4} ,P_{0} = n_{5} ,A_{0} = n_{6} ,H_{0} = n_{7} ,R_{0} = n_{8} ,F_{0} = n_{9} .$$

where ${}^{{{{}{}}c}}D^{\alpha } 0 \le \alpha \le 1$ represent Fractional order derivative of Caputo and $\alpha$ indicate time-fractional derivative. By taking Laplace transform on the system given in (12), we get

13 $$\left. {\begin{array}{*{20}l} {L\left\{ {{}^{c}D^{{\alpha_{1} }} S(t)} \right\} = L\left\{ {\pi (1 - \rho ) - K_{1} I(t)S(t) + K_{2} H(t)S(t) + K_{3} P(t)S(t) + \omega V(t)} \right\},} \hfill \\ {L\left\{ {{}^{c}D^{{\alpha_{2} }} V(t)} \right\} = L\left\{ {\pi \rho - (\omega + \delta_{v} )V(t)} \right\},} \hfill \\ {L\left\{ {{}^{c}D^{{\alpha_{3} }} E(t)} \right\} = L\left\{ {K_{1} I(t)S(t) + K_{2} H(t)S(t) + K_{3} P(t)S(t) - \kappa E(t)} \right\},} \hfill \\ {L\left\{ {{}^{c}D^{{\alpha_{4} }} I(t)} \right\} = L\left\{ {\kappa \rho_{1} E(t) - (\gamma_{a} + \gamma_{1} )I(t) - \delta_{1} I(t)} \right\},} \hfill \\ {L\left\{ {{}^{c}D^{{\alpha_{5} }} P(t)} \right\} = L\left\{ {\kappa \rho_{2} E(t) - (\gamma_{a} + \gamma_{1} )P(t) - \delta_{1} P(t)} \right\},} \hfill \\ {L\left\{ {{}^{c}D^{{\alpha_{6} }} A(t)} \right\} = L\left\{ {\kappa (1 - \rho_{1} - \rho_{2} )E(t)} \right\},} \hfill \\ {L\left\{ {{}^{c}D^{{\alpha_{7} }} H(t)} \right\} = L\left\{ {\gamma_{a} (I(t) + P(t)) - \gamma_{r} H(t) - \delta_{h} H(t)} \right\},} \hfill \\ {L\left\{ {{}^{c}D^{{\alpha_{8} }} R(t)} \right\} = L\left\{ {\gamma_{1} (I(t) + P(t)) - \gamma_{r} H(t)} \right\},} \hfill \\ {L\left\{ {{}^{c}D^{{\alpha_{9} }} F(t)} \right\} = L\left\{ {\delta_{1} I(t) + \delta_{p} P(t) + \delta_{h} H(t) + \delta_{v} V(t)} \right\}.} \hfill \\ \end{array} } \right\}$$

By definition of Laplace transform, we obtain

14 $$\left. {\begin{array}{*{20}l} {S^{{\alpha_{1} }} S(t) - S^{{\alpha_{1} - 1}} S(0) = L\left\{ {\pi (1 - \rho ) - K_{1} I(t)S(t) + K_{2} H(t)S(t) + K_{3} P(t)S(t) + \omega V(t)} \right\},} \hfill \\ {S^{{\alpha_{2} }} V(t) - S^{{\alpha_{2} - 1}} V(0) = L\left\{ {\pi \rho - (\omega + \delta_{v} )V(t)} \right\},} \hfill \\ {S^{{\alpha_{3} }} E(t) - S^{{\alpha_{3} - 1}} E(0) = L\left\{ {K_{1} I(t)S(t) + K_{2} H(t)S(t) + K_{3} P(t)S(t) - \kappa E(t)} \right\},} \hfill \\ {S^{{\alpha_{4} }} I(t) - S^{{\alpha_{4} - 1}} I(0) = L\left\{ {\kappa \rho_{1} E(t) - (\gamma_{a} + \gamma_{1} )I(t) - \delta_{1} I(t)} \right\},} \hfill \\ {S^{{\alpha_{5} }} P(t) - S^{{\alpha_{5} - 1}} P(0) = L\left\{ {\kappa \rho_{2} E(t) - (\gamma_{a} + \gamma_{1} )P(t) - \delta_{1} P(t)} \right\},} \hfill \\ {S^{{\alpha_{6} }} A(t) - S^{{\alpha_{6} - 1}} A(0) = L\left\{ {\kappa (1 - \rho_{1} - \rho_{2} )E(t)} \right\},} \hfill \\ {S^{{\alpha_{7} }} H(t) - S^{{\alpha_{7} - 1}} H(0) = L\left\{ {\gamma_{a} (I(t) + P(t)) - \gamma_{r} H(t) - \delta_{h} H(t)} \right\},} \hfill \\ {S^{{\alpha_{18} }} R(t) - S^{{\alpha_{8} - 1}} R(0) = L\left\{ {\gamma_{1} (I(t) + P(t)) - \gamma_{r} H(t)} \right\},} \hfill \\ {S^{{\alpha_{9} }} F(t) - S^{{\alpha_{9} - 1}} F(0) = L\left\{ {\delta_{1} I(t) + \delta_{p} P(t) + \delta_{h} H(t) + \delta_{v} V(t)} \right\}.} \hfill \\ \end{array} } \right\}$$

Assumed that, the method gives an infinite series solution such that:

15 $$\begin{aligned} & S(t) = \sum\limits_{n = 0}^{ \propto } {S_{n} } ,V(t) = \sum\limits_{n = 0}^{ \propto } {V_{n} } ,E(t) = \sum\limits_{n = 0}^{ \propto } {E_{n} ,} .P(t) = \sum\limits_{n = 0}^{ \propto } {P_{n} } ,I(t) = \sum\limits_{n = 0}^{ \propto } {I_{n} } , \\ & A(t) = \sum\limits_{n = 0}^{ \propto } {A_{n} } ,H(t) = \sum\limits_{n = 0}^{ \propto } {H_{n} } ,R(t) = \sum\limits_{n = 0}^{ \propto } {R_{n} } ,F(t) = \sum\limits_{n = 0}^{ \propto } {F_{n} } , \\ \end{aligned}$$

The nonlinear term involved are $I(t)S(t)$, $H(t)S(t)$, $P(t)S(t)$ can be written as

16 $$I(t)S(t) = \sum\limits_{n = 0}^{ \propto } {M_{n} } ,H(t)S(t) = \sum\limits_{n = 0}^{ \propto } {Q_{n} } \;{\text{and}}\;P(t)S(t) = \sum\limits_{n = 0}^{ \propto } {W_{n} }$$

where $M_{n}$, $Q_{n}$, $W_{n}$ are Adomian polynomial given by

17 $$\begin{aligned} M_{n} & = \frac{1}{\Gamma (n + 1)}\frac{{{\text{d}}^{n} }}{{{\text{d}}t}}\left[ {\sum\limits_{k = o}^{n} {^{{}} } \lambda^{k} I_{K} \sum\limits_{k = 0}^{n} {\lambda^{n} } S_{k} } \right]\lambda \\ Q_{n} & = \frac{1}{\Gamma (n + 1)}\frac{{{\text{d}}^{n} }}{{{\text{d}}t}}\left[ {\sum\limits_{k = o}^{n} {^{{}} } \lambda^{k} H_{K} \sum\limits_{k = 0}^{n} {\lambda^{n} } S_{k} } \right]\lambda \\ W_{n} & = \frac{1}{\Gamma (n + 1)}\frac{{{\text{d}}^{n} }}{{{\text{d}}t}}\left[ {\sum\limits_{k = o}^{n} {^{{}} } \lambda^{k} P_{K} \sum\limits_{k = 0}^{n} {\lambda^{n} } S_{k} } \right]\lambda \\ \end{aligned}$$

Substitute Eqs. (15), (16) and (17) into (14), we obtain:

18 $$\left. {\begin{array}{*{20}l} {S(t) = \frac{{n_{1} }}{S} + \frac{1}{{S^{{\alpha_{1} }} }}L\left\{ {\pi (1 - \rho ) - K_{1} M_{n} + K_{2} Q_{n} + K_{3} W_{n} + \omega V_{n} } \right\},} \hfill \\ {V(t) = \frac{{n_{2} }}{S} + \frac{1}{{S^{{\alpha_{2} }} }}L\left\{ {\pi \rho - (\omega + \delta_{v} )V_{n} } \right\},} \hfill \\ {E(t) = \frac{{n_{3} }}{S} + \frac{1}{{S^{{\alpha_{3} }} }}L\left\{ {K_{1} M_{n} + K_{2} Q_{n} + K_{3} W_{n} - \kappa E_{n} } \right\},} \hfill \\ {I(t) = \frac{{n_{4} }}{S} + \frac{1}{{S^{{\alpha_{4} }} }}L\left\{ {\kappa \rho_{1} E_{n} - (\gamma_{a} + \gamma_{1} )I_{n} - \delta_{1} I_{n} } \right\},} \hfill \\ {P(t) = \frac{{n_{5} }}{S} + \frac{1}{{S^{{\alpha_{5} }} }}L\left\{ {\kappa \rho_{2} E_{n} - (\gamma_{a} + \gamma_{1} )P_{n} - \delta_{1} P_{n} } \right\},} \hfill \\ {A(t) = \frac{{n_{6} }}{S} + \frac{1}{{S^{{\alpha_{6} }} }}L\left\{ {\kappa (1 - \rho_{1} - \rho_{2} )E_{n} } \right\},} \hfill \\ {H(t) = \frac{{n_{7} }}{S} + \frac{1}{{S^{{\alpha_{7} }} }}L\left\{ {\gamma_{a} (I_{n} + P_{n} ) - \gamma_{r} H_{n} - \delta_{h} H_{n} } \right\},} \hfill \\ {R(t) = \frac{{n_{8} }}{S} + \frac{1}{{S^{{\alpha_{8} }} }}L\left\{ {\gamma_{1} (I_{n} + P_{n} ) - \gamma_{r} H_{n} } \right\},} \hfill \\ {F(t) = \frac{{n_{9} }}{S} + \frac{1}{{S^{{\alpha_{9} }} }}L\left\{ {\delta_{1} I_{n} + \delta_{p} P_{n} + \delta_{h} H_{n} + \delta_{v} V_{n} } \right\}.} \hfill \\ \end{array} } \right\}$$

To obtain the solution of each compartment, we iterate term in Eq. (18). Thus, taking the Laplace transform inverse gives General formula for the model:

19 $$\left. {\begin{array}{*{20}l} {\sum\limits_{n = 0}^{\infty } {S_{n + 1} } (t) = L^{ - 1} \left[ {\frac{1}{{S^{{\alpha_{1} }} }}L\left\{ {\pi (1 - \rho ) - \beta \frac{{M_{n} }}{N} - l\beta \frac{{Q_{n} }}{N} - \beta^{^{\prime}} \frac{{W_{n} }}{N} + \omega V_{n} } \right\}} \right],} \hfill \\ {\sum\limits_{n = 0}^{\infty } {V_{n + 1} } (t) = L^{ - 1} \left[ {\frac{1}{{S^{{\alpha_{2} }} }}L\left\{ {\pi \rho - (\omega + \delta_{v} )V_{n} } \right\}} \right],} \hfill \\ {\sum\limits_{n = 0}^{\infty } {E_{n + 1} } (t) = L^{ - 1} \left[ {\frac{1}{{S^{{\alpha_{3} }} }}L\left\{ {\beta \frac{{M_{n} }}{N} + l\beta \frac{{Q_{n} }}{N} + \beta^{^{\prime}} \frac{{W_{n} }}{N} - \kappa E_{n} } \right\}} \right],} \hfill \\ {\sum\limits_{n = 0}^{\infty } {I_{n + 1} } (t) = L^{ - 1} \left[ {\frac{1}{{S^{{\alpha_{4} }} }}L\left\{ {\kappa \rho_{1} E_{n} - (\gamma_{a} + \gamma_{1} )I_{n} - \delta_{1} I_{n} } \right\}} \right],} \hfill \\ {\sum\limits_{n = 0}^{\infty } {P_{n + 1} } (t) = L^{ - 1} \left[ {\frac{1}{{S^{{\alpha_{5} }} }}L\left\{ {\kappa \rho_{2} E_{n} - (\gamma_{a} + \gamma_{1} )P_{n} - \delta_{1} P_{n} } \right\}} \right],} \hfill \\ {\sum\limits_{n = 0}^{\infty } {A_{n + 1} } (t) = L^{ - 1} \left[ {\frac{1}{{S^{{\alpha_{6} }} }}L\left\{ {\kappa (1 - \rho_{1} - \rho_{2} )E_{n} } \right\}} \right],} \hfill \\ {\sum\limits_{n = 0}^{\infty } {H_{n + 1} } (t) = L^{ - 1} \left[ {\frac{1}{{S^{{\alpha_{7} }} }}L\left\{ {\gamma_{a} (I_{n} + P_{n} ) - \gamma_{r} H_{n} - \delta_{h} H_{n} } \right\}} \right],} \hfill \\ {\sum\limits_{n = 0}^{\infty } {R_{n + 1} } (t) = L^{ - 1} \left[ {\frac{1}{{S^{{\alpha_{8} }} }}L\left\{ {\gamma_{1} (I_{n} + P_{n} ) - \gamma_{r} H_{n} } \right\}} \right],} \hfill \\ {\sum\limits_{n = 0}^{\infty } {F_{n + 1} } (t) = L^{ - 1} \left[ {\frac{1}{{S^{{\alpha_{9} }} }}L\left\{ {\delta_{1} I_{n} + \delta_{p} P_{n} + \delta_{h} H_{n} + \delta_{v} V_{n} } \right\}} \right].} \hfill \\ \end{array} } \right\}$$

The followings were obtained from (19);

$$S_{0} = n_{1} ,V_{0} = n_{2,} ,E_{0} = n_{3} ,I_{0} = n_{4} ,P_{0} = n_{5} ,A_{0} = n_{6} ,H_{0} = n_{7} ,R_{0} = n_{8} ,F_{0} = n_{9} .$$

$$S_{1} = \left( {\pi (1 - \rho ) - K_{1} n_{4} n_{1} - K_{2} n_{7} n_{1} - K_{3} n_{5} n_{i} + \omega n_{2} } \right)\frac{{t^{{\alpha_{1} }} }}{{\Gamma (\alpha_{1} + 1)}}.$$

$$V_{1} = \left( {\pi \rho - (\omega + \delta_{v} )n_{2} } \right)\frac{{t^{{\alpha_{2} }} }}{{\Gamma (\alpha_{2} + 1)}}.$$

$$E_{1} = \left( {K_{1} n_{4} n_{1} + K_{2} n_{7} n_{1} + K_{3} n_{5} n_{1} - \kappa n_{2} } \right)\frac{{t^{{\alpha_{3} }} }}{{\Gamma (\alpha_{3} + 1)}}.$$

$$I_{1} = (\kappa \rho_{1} n_{3} - (\gamma_{a} + \gamma_{1} )n_{4} - \delta_{1} n_{4} )\frac{{t^{{\alpha_{4} }} }}{{\Gamma (\alpha_{4} + 1)}}.$$

$$P_{1} = \left( {\kappa \rho_{2} n_{3} - (\gamma_{a} + \gamma_{1} )n_{5} - \delta_{1} n_{5} } \right)\frac{{t^{{\alpha_{5} }} }}{{\Gamma (\alpha_{5} + 1)}}.$$

$$A_{1} = \left( {\kappa (1 - \rho_{1} - \rho_{2} )n_{3} } \right)\frac{{t^{\alpha } }}{{\Gamma (\alpha_{6} + 1)}}.$$

$$H_{1} = \left( {\gamma_{a} (n_{4} + n_{5} ) - \gamma_{r} n - \delta_{h} n_{7} } \right)\frac{{t^{{\alpha_{7} }} }}{{\Gamma (\alpha_{7} + 1)}}.$$

$$R_{1} = \left( {\gamma_{1} (n_{4} + n_{5} ) - \gamma_{r} n_{7} } \right)\frac{{t^{{\alpha_{8} }} }}{{\Gamma (\alpha_{8} + 1)}}.$$

$$F_{1} = \left( {\delta_{1} n_{4} + \delta_{p} n_{5} + \delta_{h} n_{7} + \delta_{v} n_{2} } \right)\frac{{t^{{\alpha_{9} }} }}{{\Gamma (\alpha_{9} + 1)}}.$$

$$\begin{aligned} S_{2} & = \left[ {\pi (1 - \rho ) - K_{1} \left[ {(n_{4} )(\pi (1 - \rho ) - K_{1} n_{4} n_{1} - K_{2} n_{7} n_{1} - K_{3} n_{5} n_{i} + \omega n_{2} )\frac{{t^{{2\alpha_{1} }} }}{{\Gamma (2\alpha_{1} + 1)}}} \right.} \right. \\ & \quad \left. { + (n_{1} )(\kappa \rho_{1} n_{3} - (\gamma_{a} + \gamma_{1} )n_{4} - \delta_{1} n_{4} )\frac{{t^{{\alpha_{1} + \alpha_{4} }} }}{{\Gamma (\alpha_{1} + \alpha_{4} + 1)}}} \right] \\ & \quad - K_{2} \left[ {(n_{7} )(\pi (1 - \rho ) - K_{1} n_{4} n_{1} - K_{2} n_{7} n_{1} - K_{3} n_{5} n_{i} + \omega n_{2} )\frac{{t^{{2\alpha_{1} }} }}{{\Gamma (2\alpha_{1} + 1)}}} \right. \\ & \quad \left. { + (n_{1} )(\gamma_{a} (n_{4} + n_{5} ) - \gamma_{r} n_{{}} - \delta_{h} n_{7} )\frac{{t^{{\alpha_{1} + \alpha_{7} }} }}{{\Gamma (\alpha_{1} + \alpha_{7} + 1)}}} \right] \\ & \quad - K_{3} \left[ {(n_{5} )(\pi (1 - \rho ) - K_{1} n_{4} n_{1} - K_{2} n_{7} n_{1} - K_{3} n_{5} n_{i} + \omega n_{2} )\frac{{t^{{2\alpha_{1} }} }}{{\Gamma (2\alpha_{1} + 1)}}} \right. \\ & \quad \left. {\left. { + (n_{1} )(\kappa \rho_{2} n_{3} - (\gamma_{a} + \gamma_{1} )n_{5} - \delta_{1} n_{5} )\frac{{t^{{\alpha_{1} + \alpha_{5} }} }}{{\Gamma (\alpha_{1} + \alpha_{5} )}}} \right] + \omega (\pi \rho - (\omega + \delta_{v} )n_{2} )\frac{{t^{{\alpha_{1} + \alpha_{2} }} }}{{S^{{\alpha_{2} + 1}} }}} \right]. \\ \end{aligned}$$

$$V_{2} = (\pi \rho - (\omega + \delta_{v} )(\pi \rho - (\omega + \delta_{v} )n_{2} )\frac{{t^{{2\alpha_{2} }} }}{{\Gamma (2\alpha ._{2} + 1)^{{}} }}.$$

$$\begin{aligned} I_{2} & = (\kappa \rho_{1} (K_{1} n_{4} n_{1} + K_{2} n_{7} n_{1} + K_{3} n_{5} n_{1} - \kappa n_{2} )\frac{{t^{{\alpha_{3} + \alpha_{4} }} }}{{\Gamma (\alpha_{3} + \alpha_{4} + 1)}} \\ & \quad - (\gamma_{a} + \gamma_{1} )(\kappa \rho_{1} n_{3} - (\gamma_{a} + \gamma_{1} )n_{4} - \delta_{1} n_{4} )\frac{{t^{{2\alpha_{4} }} }}{{\Gamma (2\alpha_{4} + 1)}} \\ & \quad - \delta_{1} (\kappa \rho_{1} n_{3} - (\gamma_{a} + \gamma_{1} )n_{4} - \delta_{1} n_{4} )\frac{{t^{{2\alpha_{4} }} }}{{\Gamma (2\alpha_{4} + 1)}}. \\ \end{aligned}$$

$$\begin{aligned} P_{2} & = (\kappa \rho_{2} (K_{1} n_{4} n_{1} + K_{2} n_{7} n_{1} + K_{3} n_{5} n_{1} - \kappa n_{2} )\frac{{t^{{\alpha_{3} + \alpha_{5} }} }}{{\Gamma (\alpha_{3} + \alpha_{5} + 1)}} \\ & \quad - (\gamma_{a} + \gamma_{1} )(\kappa \rho_{2} n_{3} - (\gamma_{a} + \gamma_{1} )n_{5} - \delta_{1} n_{5} )\frac{{t^{{2\alpha_{5} }} }}{{\Gamma (2\alpha_{5} + 1)}} \\ & \quad - \delta_{1} (\kappa \rho_{2} n_{3} - (\gamma_{a} + \gamma_{1} )n_{5} - \delta_{1} n_{5} )\frac{{t^{{2\alpha_{5} }} }}{{\Gamma (2\alpha_{5} + 1)}}. \\ \end{aligned}$$

$$A_{2} = \kappa (1 - \rho_{1} - \rho_{2} )(K_{1} n_{4} n_{1} + K_{2} n_{7} n_{1} + K_{3} n_{5} n_{1} - \kappa n_{2} )\frac{{t^{{\alpha_{3} + \alpha_{6} }} }}{{\Gamma (\alpha_{3} + \alpha_{6} + 1)}}.$$

$$\begin{aligned} H_{2} & = \gamma_{a} ((\kappa \rho_{1} n_{3} - (\gamma_{a} + \gamma_{1} )n_{4} - \delta_{1} n_{4} )\frac{{t^{{\alpha_{4} + \alpha_{7} }} }}{{\Gamma (\alpha_{4} + \alpha_{7} + 1)}} \\ & \quad + (\kappa \rho_{2} n_{3} - (\gamma_{a} + \gamma_{1} )n_{5} - \delta_{1} n_{5} )\frac{{t^{{\alpha_{5} + \alpha_{7} }} }}{{\Gamma (\alpha_{5} + \alpha_{7} + 1)}}) \\ & \quad - \gamma_{r} (\gamma_{a} (n_{4} + n_{5} ) - \gamma_{r} n_{{}} - \delta_{h} n_{7} )\frac{{t^{{2\alpha_{7} }} }}{{\Gamma (2\alpha_{7} + 1)}} \\ & \quad - \delta_{h} (\gamma_{a} (n_{4} + n_{5} ) - \gamma_{r} n_{{}} - \delta_{h} n_{7} )\frac{{t^{{2\alpha_{7} }} }}{{\Gamma (2\alpha_{7} + 1)}}. \\ \end{aligned}$$

$$\begin{aligned} R_{2} & = \gamma_{1} \left[ {(\kappa \rho_{1} n_{3} - (\gamma_{a} + \gamma_{1} )n_{4} - \delta_{1} n_{4} )\frac{{t^{{\alpha_{4} + \alpha_{8} }} }}{{\Gamma (\alpha_{4} + \alpha_{8} + 1)}}} \right. \\ & \quad \left. { + (\kappa \rho_{2} n_{3} - (\gamma_{a} + \gamma_{1} )n_{5} - \delta_{1} n_{5} )\frac{{t^{{\alpha_{5} + \alpha_{8} }} }}{{\Gamma (\alpha_{5} + \alpha_{8} + 1)}}} \right] \\ & \quad - \gamma_{r} (\gamma_{a} (n_{4} + n_{5} ) - \gamma_{r} n_{{}} - \delta_{h} n_{7} )\frac{{t^{{\alpha_{7} + \alpha_{8} }} }}{{\Gamma (\alpha_{7} + \alpha_{8} + 1)}}. \\ \end{aligned}$$

$$\begin{aligned} F_{2} & = \delta_{v} (\pi \rho - (\omega + \delta_{v} )n_{2} )\frac{{t^{{\alpha_{2} + \alpha_{9} }} }}{{\Gamma (\alpha_{2} + \alpha_{9} + 1)}} \\ & \quad + \delta_{1} (\kappa \rho_{1} n_{3} - (\gamma_{a} + \gamma_{1} )n_{4} - \delta_{1} n_{4} )\frac{{t^{{\alpha_{4} + \alpha_{9} }} }}{{\Gamma (\alpha_{4} + \alpha_{9} + 1)}} \\ & \quad + \delta_{p} (\kappa \rho_{2} n_{3} - (\gamma_{a} + \gamma_{1} )n_{5} - \delta_{1} n_{5} )_{{}} \frac{{t^{{\alpha_{5} + \alpha_{9} }} }}{{\Gamma (\alpha_{5} + \alpha_{9} + 1)}} \\ &\quad + \delta_{h} (\gamma_{a} (n_{4} + n_{5} ) - \gamma_{r} n_{{}} - \delta_{h} n_{7} )\frac{{t^{{\alpha_{7} + \alpha_{9} }} }}{{\Gamma (\alpha_{7} + \alpha_{9} + 1)}}. \\ \end{aligned}$$

### Results

Evaluating the obtained results using the following parameters;

$$S_{0} = \frac{1879813}{{17}},\;V_{0} = 20,\;E_{0} = 0,I_{0} = 10,\;P_{0} = 1,\;A_{0} = 0,\;H_{0} = 0,\;R_{0} = 0,\;F_{0} = 0.$$

$$\beta = 2.55$$, $$l = 1.56$$, $$\beta_{1} = 7.65$$, $$\kappa = 0.25$$, $$\rho_{1} = 0.58$$, $$\rho_{2} = 0.001$$, $$\gamma_{a} = 0.94$$, $$\gamma_{1} = 0.27$$, $$\gamma_{r} = 0.5$$, $$\delta_{1} = \frac{1}{3}$$, $$\delta_{1} = \frac{1}{23}$$, $$\delta_{h} = \frac{1}{23}$$, $$K_{1} = 1.27659574510^{ - 10}$$, $$K_{2} = 1.99148936210^{ - 10}$$, $$K_{3} = 3.82978723310^{ - 10}$$, $$\omega = 2$$, $$\pi = 30$$, $$\rho = 0.5$$, $$\delta_{v} = 2$$.

We obtain the following series solution of arbitrary order:

$$S = 1.10562235310^{5} + \frac{{54.9998169t^{\alpha } }}{\Gamma (\alpha + 1)} + \frac{{92.00000233t^{2\alpha } }}{\Gamma (2\alpha + 1)}$$

$$V = 0.05 - \frac{{31.0t^{\alpha } }}{\Gamma (\alpha + 1)} + \frac{{105.80t^{2\alpha } }}{\Gamma (2\alpha + 1)}$$

$$E = \frac{{0.0001835111565t^{\alpha } }}{\Gamma (\alpha + 1)} + \frac{{0.0001252054271t^{2\alpha } }}{\Gamma (2\alpha + 1)} - \frac{{0.0001769440349t^{2\alpha } }}{\Gamma (2\alpha + 1)}$$

$$I = 10 - \frac{{12.53478261t^{\alpha } }}{\Gamma (\alpha + 1)} + \frac{{15.18599669t^{2\alpha } }}{\Gamma (2\alpha + 1)}$$

$$P = 1 - \frac{{1.253478261t^{\alpha } }}{\Gamma (\alpha + 1)} + \frac{{1.571207797t^{2\alpha } }}{\Gamma (2\alpha + 1)}$$

$$A = \frac{{0.00001922279364t^{2\alpha } }}{\Gamma (2\alpha + 1)}$$

$$H = \frac{{0.94t^{\alpha } }}{\Gamma (\alpha + 1)} - \frac{{8.240530432t^{2\alpha } }}{\Gamma (2\alpha + 1)}$$

$$R = \frac{{2.97t^{\alpha } }}{\Gamma (\alpha + 1)} - \frac{{4.192830436t^{2\alpha } }}{\Gamma (2\alpha + 1)}$$

$$F = \frac{{6.478260870t^{\alpha } }}{\Gamma (\alpha + 1)} - \frac{{21.43971644t^{2\alpha } }}{\Gamma (2\alpha + 1)}$$

### Numerical simulation

To analyze the impact of Caputo fractional order in the model’s compartment, we conduct numerical simulation by varying the order at $\alpha = 0.75$, $\alpha = 0.85$ and $\alpha = 1$. The outcome of simulation process is presented graphically and discussed.

## Discussion

Figures 1, 2, 3, 4, 5, 6, 7, 8 and 9 illustrate that the fractional order Corona Virus Disease model has a larger degree of freedom than a traditional derivative-based model. Another interesting point to note is that we predicted relatively low starting values; therefore we used a short time period.

**Fig. 1:**
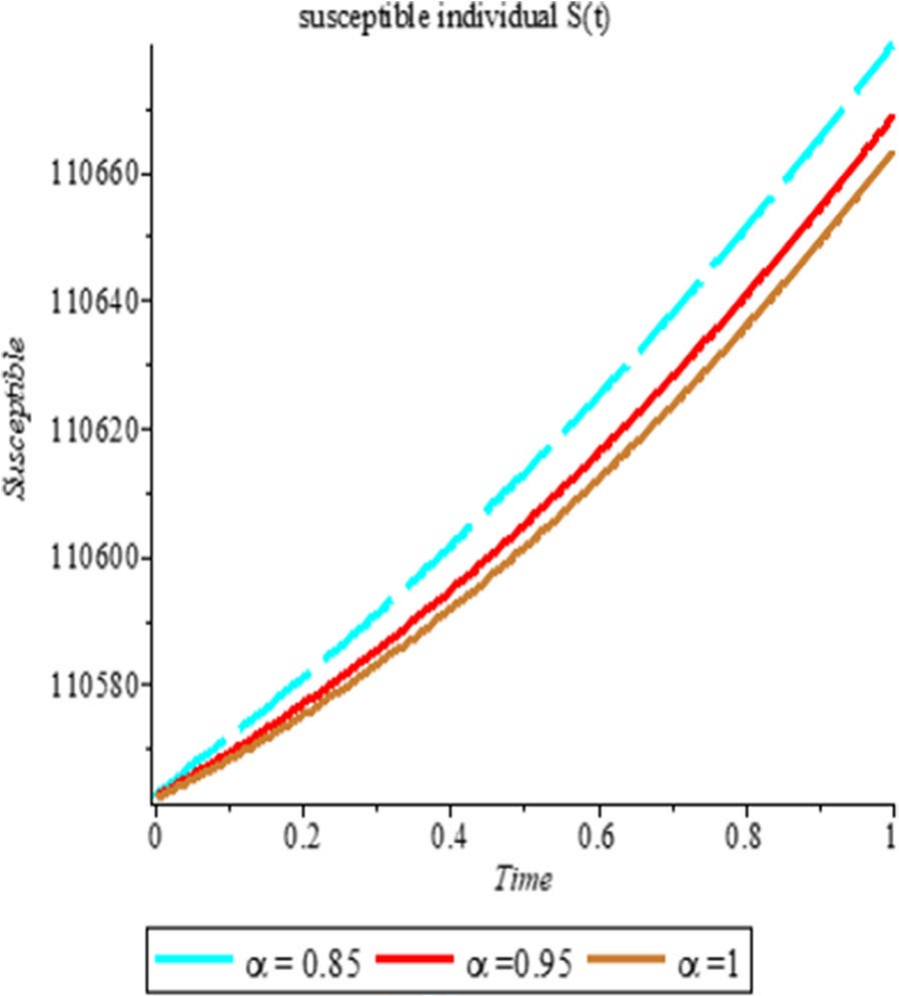
Plot shows the behavior of *S*(*t*) at different values of fractional order α

**Fig. 2:**
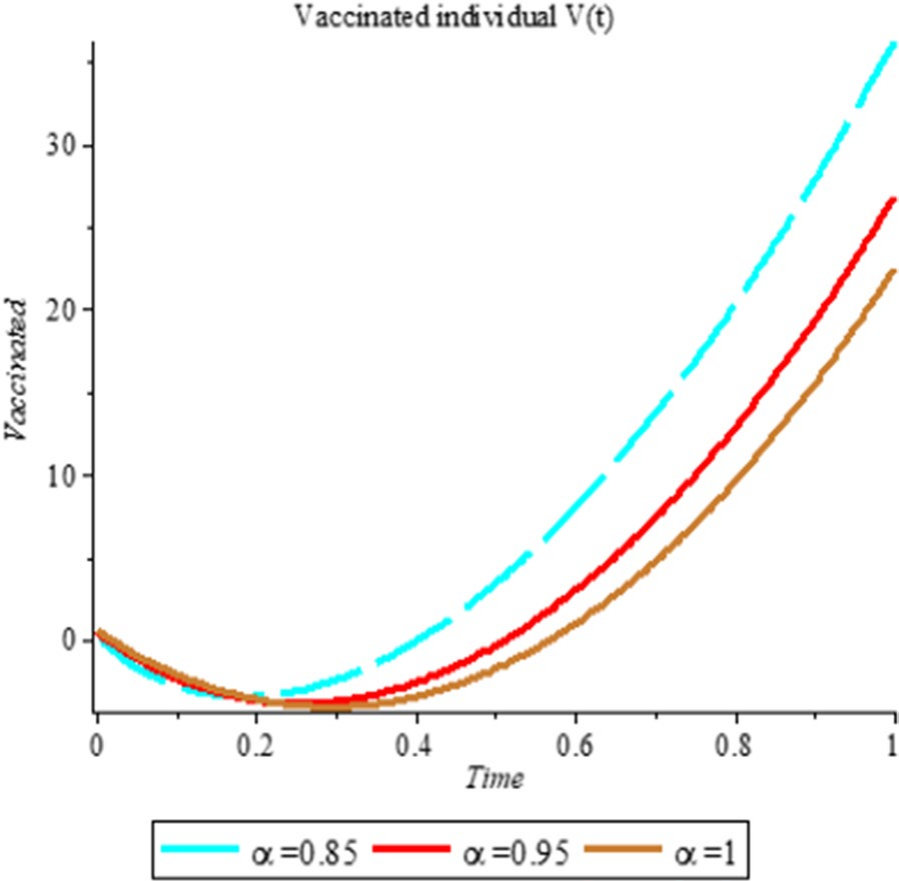
Plot shows the behavior of *V*(*t*) at different values of fractional order α

**Fig. 3:**
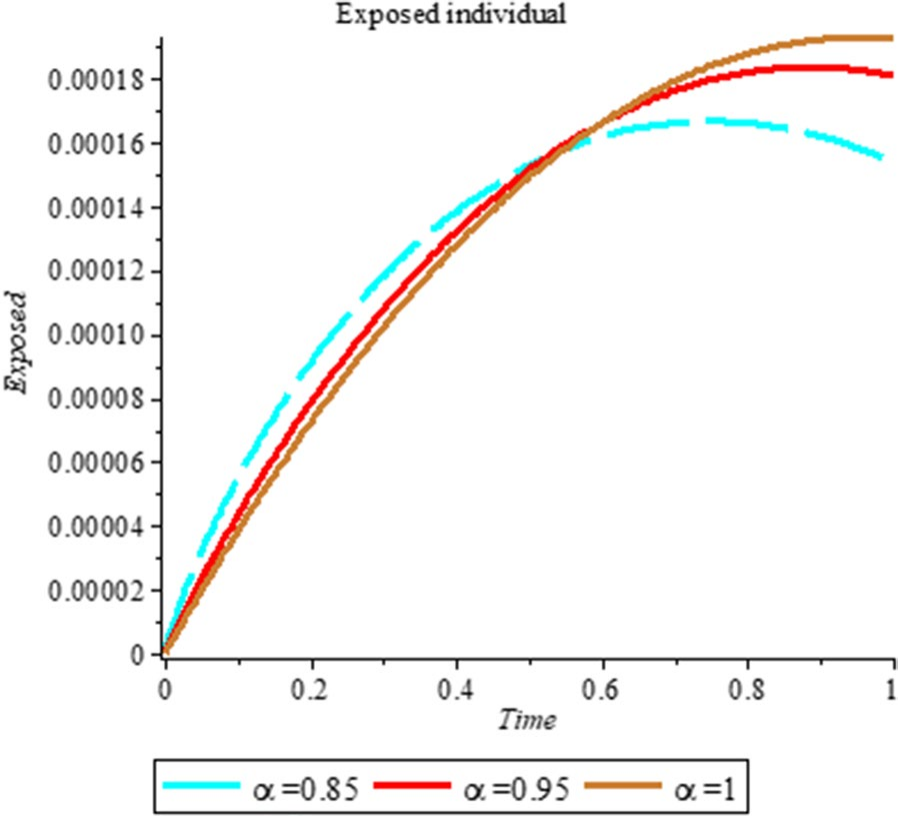
Plot shows the behavior of *E*(*t*) at different values of fractional order α

**Fig. 4:**
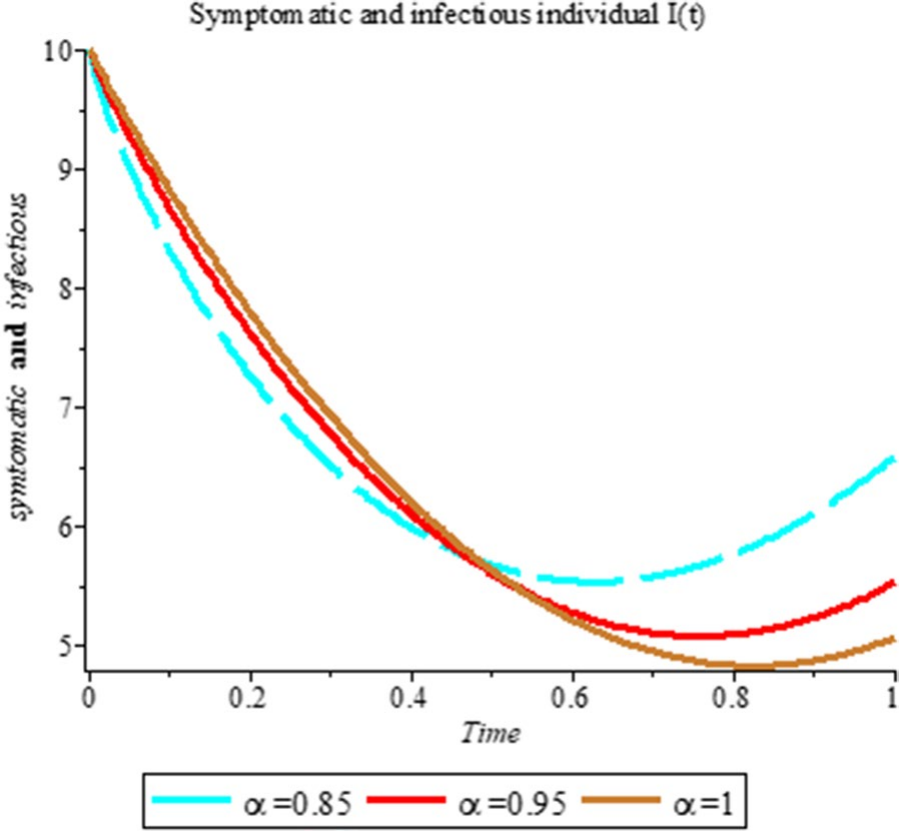
Plot shows the behavior of *I*(*t*) at different values of fractional order α

**Fig. 5:**
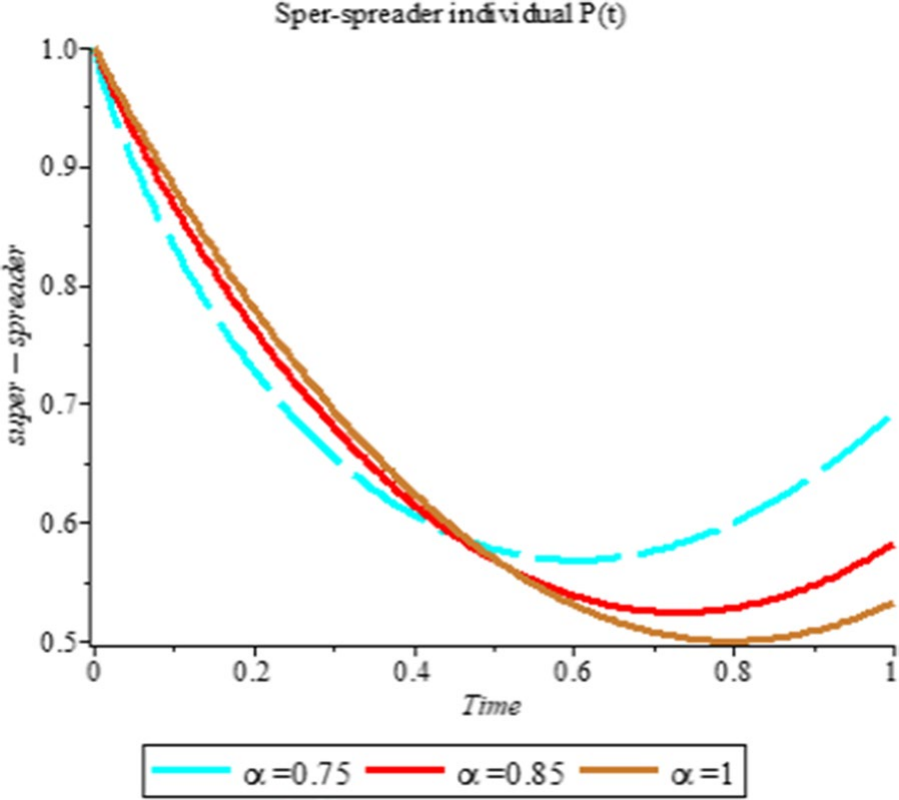
Plot shows the behavior of *P*(*t*) at different values of fractional order α

**Fig. 6:**
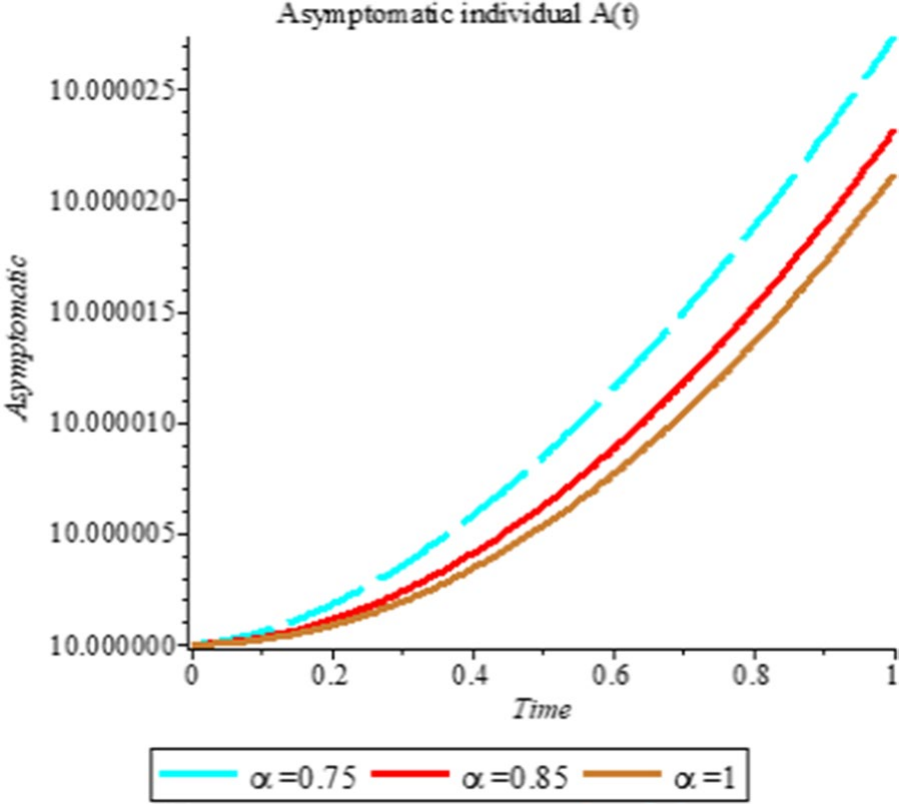
Plot shows the behavior of *A*(*t*) at different values of fractional order α

**Fig. 7:**
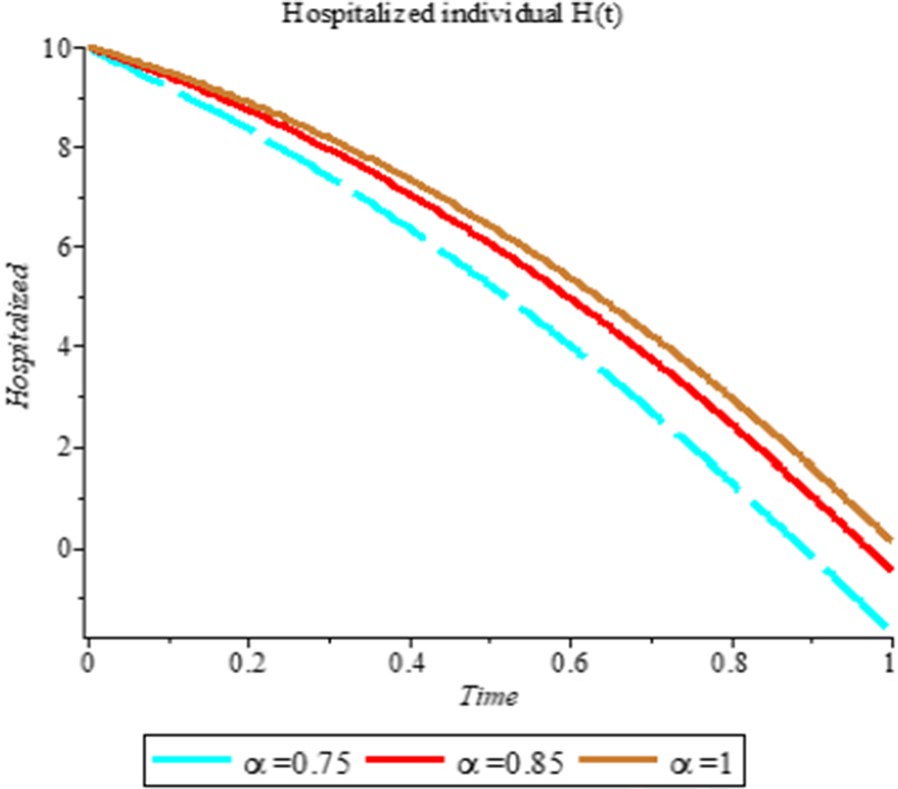
Plot shows the behavior of *H*(*t*) at different values of fractional order α

**Fig. 8:**
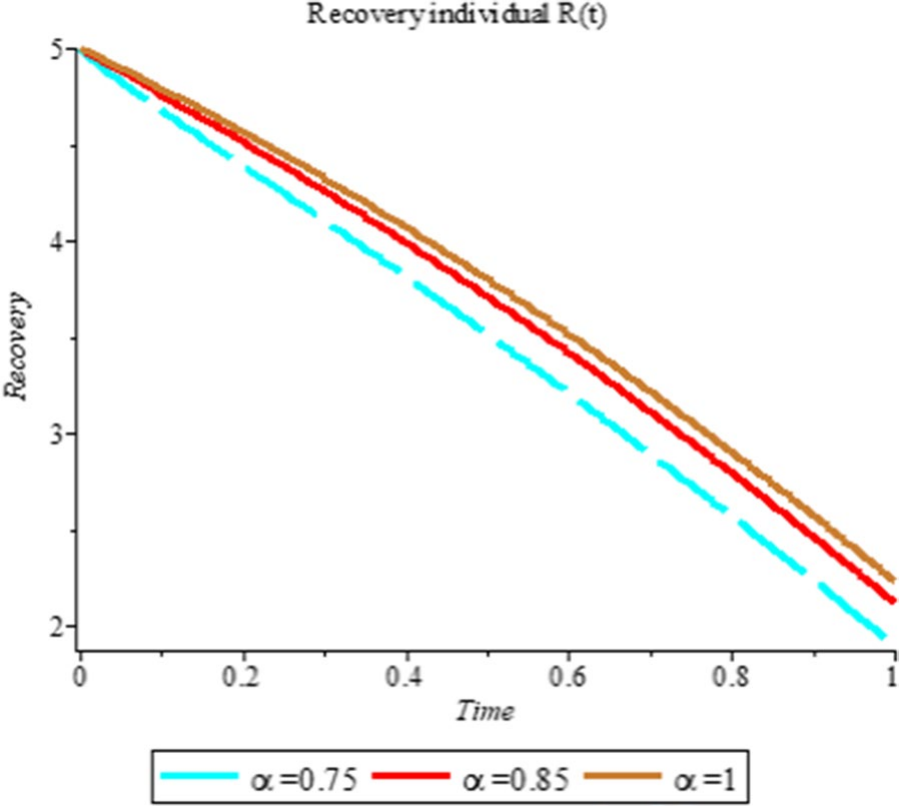
Plot shows the behavior of *R*(*t*) at different values of fractional order α

**Fig. 9:**
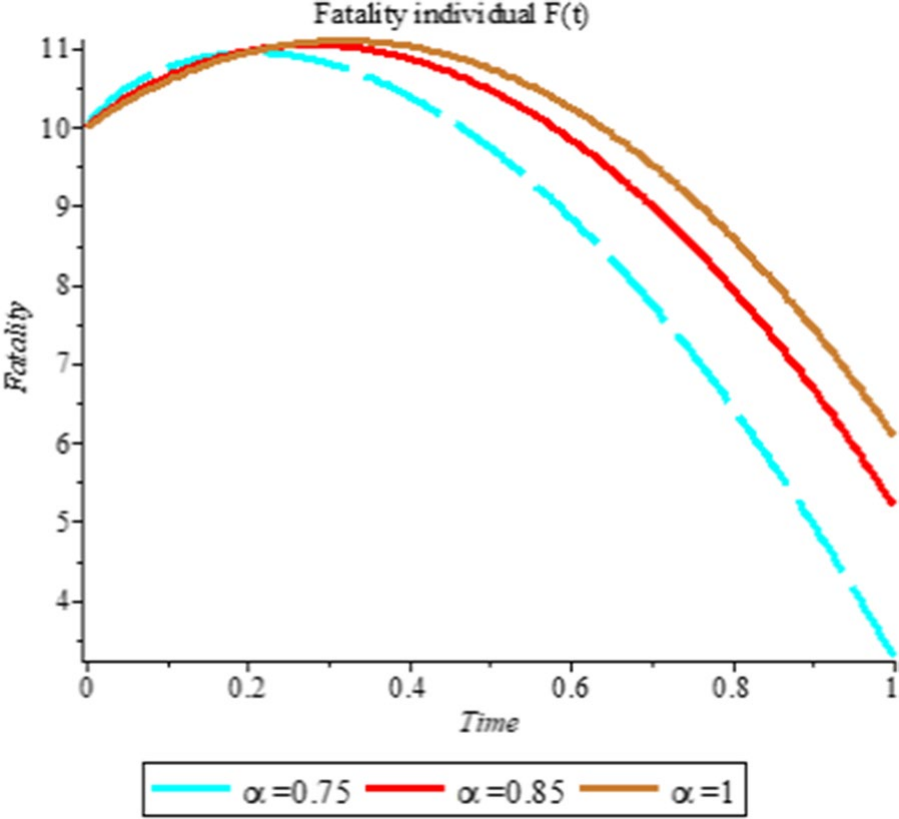
Plot shows the behavior of *F*(*t*) at different values of fractional order α

**Fig. 10:**
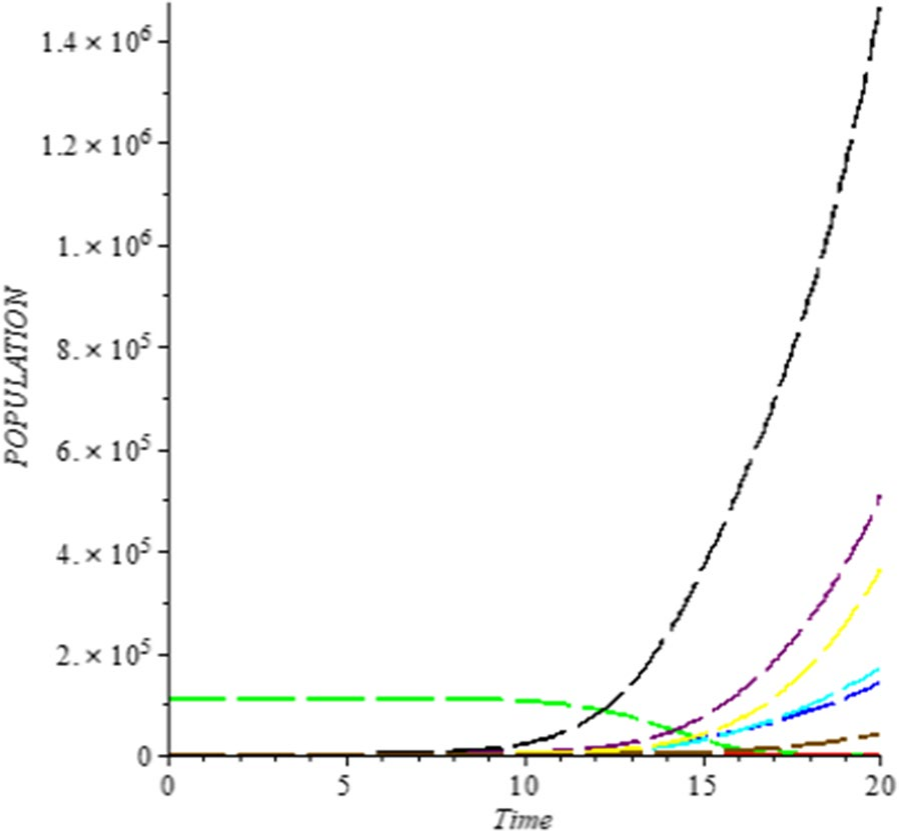
Displays the effect of *k* on *S*, *V*, *E*, *I*, *P*, *A*, *H*, *R*, *F*

**Fig. 11:**
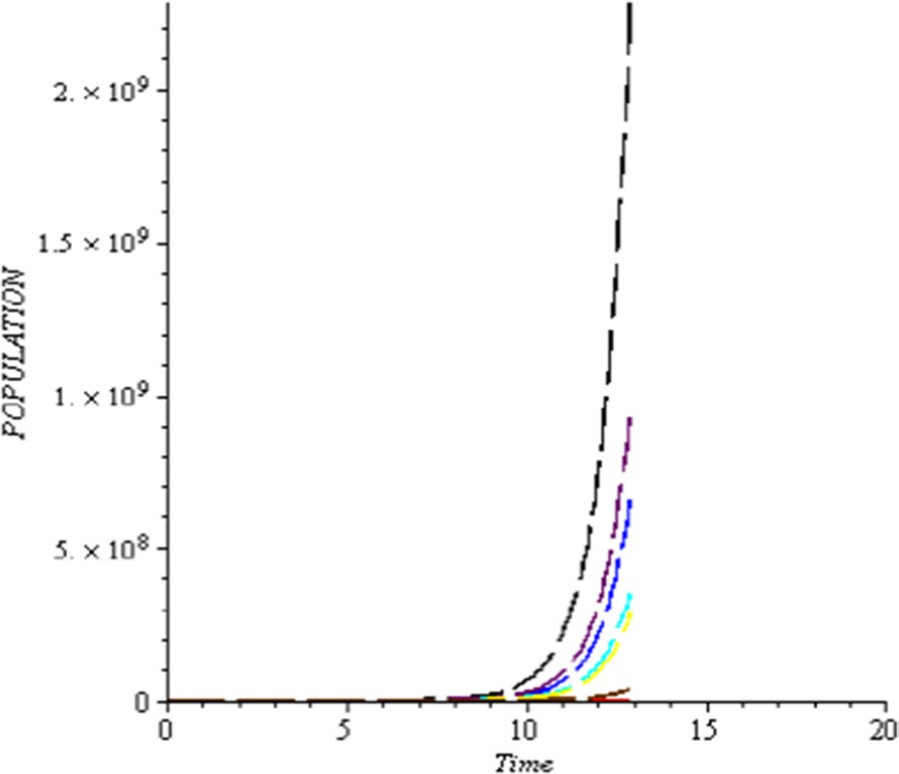
“Effect of *k*” (Rate at which individual leave the exposed class on *S*, *V*, *E*, *I*, *P*, *A*, *H*, *R*, *F*)

**Fig. 12:**
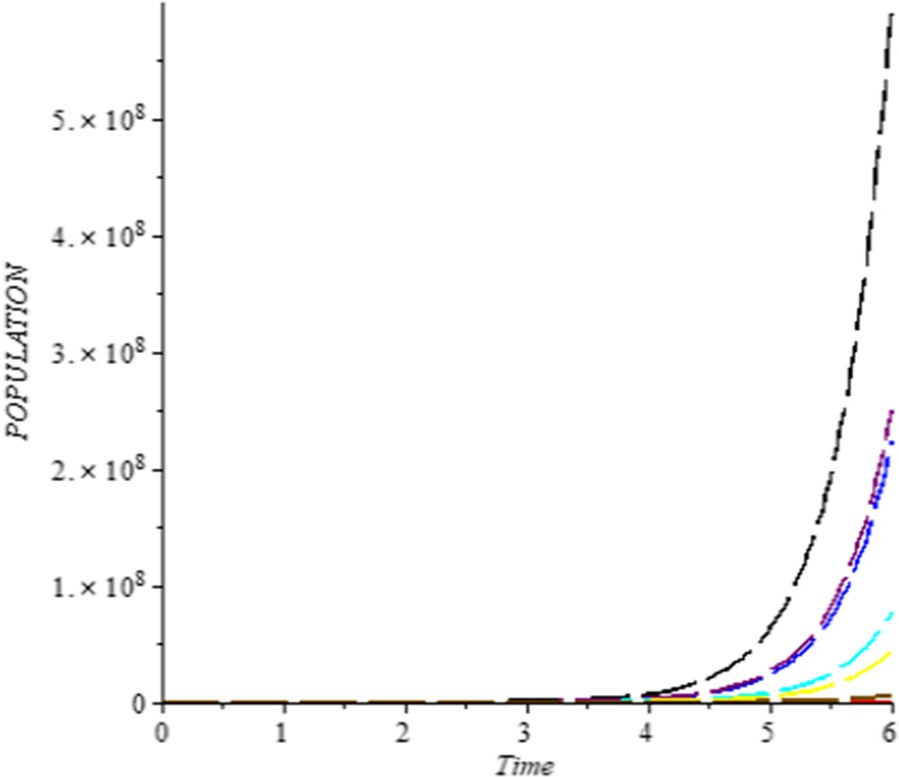
Effect of *k* (Rate at which individual leave the exposed class on *S*, *V*, *E*, *I*, *P*, *A*, *H*, *R*, *F*)

As shown in Figs. 3, 7, 8 and 9, the starting data for longer time intervals should be sufficiently large to ensure that the population under consideration is not negative. Figures 10, 11 and 12 depict the combined plots of the solutions of *S*(*t*), *V*(*t*), *E*(*t*), *I*(*t*), *P*(*t*), *A*(*t*), *H*(*t*), *R*(*t*), *F*(*t*), and *k* (the rate at which a on each compartment). Figure 10 shows that the vulnerable population has decreased significantly in susceptible individual and significant increase on other compartment. Another observation from Figs. 11 and 12 is that assuming the initial value of (*k*) is large then small interval time used. More so, all compartments take the curves from zero increase significant on the slopes.

## Conclusion

In a similar but different study presented in [10], the numerical solution of fractional order smoking model was obtained via Laplace Adomian decomposition method. In their results, they discussed that the fractional order model has more degree of freedom and therefore can be varied to get various responses of the different compartments of their model. They used small interval of time to avoid negative population of the classes which will not be realistic. They stated that for larger time interval, the initial data should be taken large so that the concerned population may not be negative. In our Research, we verified the existence and uniqueness of the model and applied this same Laplace Adomian decomposition method which proved to be effective in computing the solution of the proposed mathematical models as it produced an unconditionally convergent solution potent of capturing the dynamics of coronavirus disease as seen in our numerical simulation. As observed in some of the presented graphs, negative values of the population will be observed if time is increased. As a result, we equally postulate that larger values of initial data should be used for further research if longer time period will be investigated. More so, the validity of the model will be established if real data are applied to conduct the numerical simulations as there are high possibilities to examine the behavior of each classes of the model due to potential increase in time.

### Recommendations

Although the Caputo fractional order derivative and Laplace Adomian decomposition method proved effective and potent for understanding the dynamics of transmission and computing the solution of the proposed system in this study, we recommend that interested researchers in field extends the theory of fractional calculus to the advance the research by numerically simulating the effect of other fractional derivatives such as the Atangana–Baleanu and Caputo–Fabrizio using other numeral numerical methods such as the homotopy perturbation method or the Homotopy analysis method on the mathematical model using real data. Also further studies could be carried out by splitting the vaccination compartment into two to analyze the effect of first and second doses in the population, respectively. The first dose was used in this study, whereas the second dose can be used in future studies. In disease control, the vaccination compartment is crucial.

## Data Availability

Not applicable.

## Abbreviations

Covid-19: Coronavirus disease 2019
SVEIPAHRF: Susceptible-vaccination-exposed-symptomatic-super-spreader-infectious-hospitalize-recovered-death
LADM: Laplace Adomian decomposition method
R_0_: Reproduction number
